# Emergent Spatial Patterns of Excitatory and Inhibitory Synaptic Strengths Drive Somatotopic Representational Discontinuities and their Plasticity in a Computational Model of Primary Sensory Cortical Area 3b

**DOI:** 10.3389/fncom.2016.00072

**Published:** 2016-07-25

**Authors:** Kamil A. Grajski

**Affiliations:** NuroSci, LLC.West Palm Beach, FL, USA

**Keywords:** area 3b, somatosensory cortex, syndactyly, inhibitory synaptic plasticity, somatotopy, neuroplasticity

## Abstract

Mechanisms underlying the emergence and plasticity of representational discontinuities in the mammalian primary somatosensory cortical representation of the hand are investigated in a computational model. The model consists of an input lattice organized as a three-digit hand forward-connected to a lattice of cortical columns each of which contains a paired excitatory and inhibitory cell. Excitatory *and* inhibitory synaptic plasticity of feedforward *and* lateral connection weights is implemented as a simple covariance rule and competitive normalization. Receptive field properties are computed independently for excitatory and inhibitory cells and compared within and across columns. Within digit representational zones intracolumnar excitatory and inhibitory receptive field extents are concentric, single-digit, small, and unimodal. Exclusively in representational boundary-adjacent zones, intracolumnar excitatory and inhibitory receptive field properties *diverge*: excitatory cell receptive fields are single-digit, small, and unimodal; and the paired inhibitory cell receptive fields are bimodal, double-digit, and large. In simulated syndactyly (webbed fingers), boundary-adjacent intracolumnar receptive field properties reorganize to within-representation type; divergent properties are *reacquired* following syndactyly release. This study generates testable hypotheses for assessment of cortical laminar-dependent receptive field properties and plasticity within and between cortical representational zones. For computational studies, present results suggest that concurrent excitatory and inhibitory plasticity may underlie novel emergent properties.

## Introduction

Representational discontinuity is a striking feature of the normal somatotopic map of the hand in primary sensory cortical area 3b in adult owl monkeys (Merzenich et al., 1978; Merzeznich et al., 1987; Kaas et al., 1979; Sur, 1980; Sur et al., 1980; Merzenich et al., 1990; Recanzone et al., 1992). Excitatory receptive fields empirically derived in cortical layer IV samples spaced as closely as 50–100 μm can have profoundly different extents on the skin surface. Within representational zones excitatory receptive fields typically display a remarkable continuity: small to medium-sized, unimodal excitatory receptive fields overlap in smooth progressions over the skin surface. Between representational zones “step changes” were reported including those between adjacent digits, between glabrous and hairy skin, between palmar pads and digits, and between the face and digits, among others.

The purpose of this computational study is to explain—at the level of spatial patterns of adapted synaptic weights in excitatory and inhibitory model neurons—the emergence and reorganization of somatotopic representational continuities and discontinuities. The model system is that of area 3b in adult owl monkeys and its reorganization subject to digital syndactyly and release (Allard et al., 1991).

This study shares features in common with previous studies. First, of interest is the contribution of the interplay between excitation and inhibition (e.g., lateral or surround inhibition) in determining individual and collective excitatory receptive field properties. Second the basic model unit is that of a cortical column that consists of excitatory and inhibitory cell(s) with characteristic interconnectivity, including mutual excitatory-excitatory cell connections and recurrent inhibition.

This study is unique in two ways. First, all synaptic types are adapted subject to a simple covariance rule. These include synapses between sensory afferents to cortical excitatory cells, lateral cortical excitatory to excitatory cells, lateral cortical excitatory to inhibitory cells, and lateral cortical inhibitory to excitatory cells. Adaptation of the excitatory to excitatory connection type is Hebbian-like: spiking rate activity in the former is facilitative of changes in spiking rate in the latter. The same holds for the excitatory to inhibitory connection type. Since the inhibitory to excitatory connection type is facilitative of *not* firing action potentials in the latter due to action potential firing by the former the adaptation rule is not strictly Hebbian, but is covariance-driven. Other than an initial local random weight distribution, there is no external overlay of synaptic patterning, for example, to effect details of lateral inhibition. Second, the study reports the results of separate analyses of individual and collective receptive field properties observed in the population of excitatory cells (excitatory receptive fields) *and* in the population of inhibitory cells (inhibitory receptive fields). This enables a more detailed understanding particularly of the role of inhibition in cortical somatotopy. Spatial patterns of synaptic strengths are observed that appear not to conform simply to lateral inhibition (at least in its most simplistic stereotypical form).

The study concludes that in the present neural network model, representational continuities and discontinuities and their reorganization are a consequence of the emergence of characteristic spatial patterns of synaptic weights in both excitatory and inhibitory neural populations. The study illustrates these spatial patterns and tracks their evolution in a simulated three-digit somatotopic map from initial random conditions, to a baseline refinement, through simulated digital syndactyly, and release from syndactyly.

The effects typically attributed to and which result as a consequence of lateral inhibition are shown to be an emergent property of neural networks whose connection weights are subject to a simple covariance adaptation rule. These may represent a novel emergent neural mechanism contributing to cortical somatotopy.

## Materials and methods

### Model

The model consists of a pair of forward connected two-dimensional lattices each containing a 45 × 45 array of nodes. An input layer (S) node consists of a single “input unit” with exclusively ascending connections to its corresponding topographically centered 7 × 7 neighborhood of cortical layer (C) nodes. There is no skin surface model and no mechanoreceptor-specific model. Individual input layer afferents are experimentally activated as ON or OFF (with additive noise). The activity on these afferents is considered to model individual and groups of fast conduction velocity low-threshold cutaneous mechanoreceptors positioned in glabrous skin (Zimmerman et al., 2014).

A cortical layer node consists of an excitatory (E) and inhibitory (I) cell pair. These are interpreted as a lumped representation of a cortical column. Cortical layer E-cells are the exclusive target of S layer ascending connections. A cortical layer E-cell makes collateral excitatory connections with itself, with a corresponding topographically centered 7 × 7 neighborhood of E-cells, and with a neighborhood of I-cells. A C layer I-cell makes collateral inhibitory connections with a corresponding topographically centered 7 × 7 neighborhood of E-cells. See Tables 1A–G (formatted as proposed in Nordlie et al., 2009). Neither axonal nor other spatial delay is modeled. Normalization of input weights enforces planar boundary conditions. Control experiments confirm that for the present settings of lateral connection spatial divergence and network sizes *N* > 20 edge effects are confined to the regions one to three nodes in from each edge. The C layer consists of 2025 columns (4050 cells) and ~400,000 synapses.

**Table 1A:** **Model Summary**.

Populations	Three: excitatory (E), inhibitory (I), external excitatory input (S).
Topology	Cartesian grids for cortical layer (C) and external input layer (S)
Connectivity	Rectangular mask; excitatory self-connection: yes; inhibitory self-connection: no; multiple connections: no.
Neuron model	Lumped RC model, average spiking rate (*r_*i*_*) proportional to membrane potential (*v_*i*_*) via sigmoidal function, g(*v*).
Synapse model	Connection weight (*w_*i, j*_ = w_*post, pre*_*)
Plasticity	Connection weight is a linear combination of exponential decay term and a term proportional to the covariance of presynaptic and postsynaptic average firing rates, and postsynaptically normalized to a population type dependent “resource” level.
Input	Fixed-size, fixed-duration, and fixed-magnitude (with additive random noise) rectangular masks presented in random positions within or across external input layer partitions to model three independent digits of a hand.
Measurements	Membrane potential; average spike rate; excitatory and inhibitory receptive fields.

**Table 1B:** **Populations**.

**Name**	**Elements**	**Size**
E	Cortical column excitatory element	*N*_E_ = 2025 (*e.g.*, 45 × 45 cortical layer grid)
I	Cortical column inhibitory element	*N*_I_ = 2025 (*e.g.*, 45 × 45 cortical layer grid)
S	Excitatory external input element	*N*_S_ = 2025 (*e.g.*, 45 × 45 input layer grid)

**Table 1C:** **Connectivity**.

**Name**	**Source**	**Target**	**Pattern**
EE	E	E	Centered local M × M grid (*M* = 7); random initial weights; no delay
IE	E	I	Centered local M × M grid (*M* = 7); random initial weights; no delay
EI	I	E	Centered local M × M grid (*M* = 7); random initial weights; no delay
ES	S	E	Centered local M × M grid (*M* = 7); random initial weights; no delay

**Table 1D:** **Lumped Neuron and Input Unit Models**.

**Type**	**Description**
Cortical column excitatory element	$v_{i}^{E}(t+1)=\alpha_{m}\;v_{i}^{E}(t)\;+\;\sum_{j}r_{j}^{S}\left(t\right)w_{ij}^{ES}(t)\;+\;\sum_{j}r_{j}^{E}\left(t\right)w_{ij}^{EE}(t)\;-\;\sum_{j}r_{j}^{I}\left(t\right)w_{ij}^{EI}(t)\;+\;\epsilon \left(t\right),$ $\alpha_{m}=\left(1-\text{ h}/\tau_{\text{m}}\right),\text{ h}=0.001,\tau_{\text{m}}=0.025$ϵ(*t*) i.i.d. uniform distribution of values between ±0.01
Cortical column inhibitory element	$v_{i}^{I}(t+1)=\alpha_{m}\;v_{i}^{I}(t)\;+\;\sum_{j}r_{j}^{E}\left(t\right)w_{ij}^{IE}(t)\;+\;\epsilon (t)$
Excitatory input layer element	$v_{i}^{S}(t+1)=\alpha_{m}\;v_{i}^{S}(t)\;+\;\delta_{i}^{S}(t)+\epsilon \left(t\right)$, $\delta_{i}^{S}-S$ unit *i* output OFF (= 0) or ON (= 1)
Spiking, r	$r=g(v)=\frac{1}{2}(\;1+tanh\left[\beta_{m}\;\left(v-\frac{1}{2}\right)\right],\;\beta_{m}=4.0$
Numerical	Fourth Order Runge-Kutta numerical integration with 1 ms step size: △*t* = *h* = 0.001.

**Table 1E:** **Synapse Model**.

Initialization	All initial weight values uniform i.i.d. Postsynaptic normalization. Planar boundary conditions. No spatial preconditioning.	
Adaptation	$w_{ij}(t+1)=\;\alpha_{w}w_{ij}(t)+\;\beta_{w}\left(t\right)r_{i}\left(t\right)r_{j}(t),$ $\;\alpha_{w}=\left(1-h/\tau_{w}\right),\tau_{w}=100\tau_{m},$ $\;\beta_{w}\left(T+1\right)=\;\alpha_{\beta }\beta_{w}\left(T\right),\;\alpha_{\beta }=0.99,\;\beta_{w}\left(T=0\right)=\left(0.00025\right),\;T\;defined\;below$(Table 1F)	
Postsynaptic Normalization	Excitatory: $\frac{1}{N_{i}}\sum_{j}w_{ij}=\;R_{w}=2.0$	Inhibitory: $\frac{1}{N_{i}}\sum_{j}w_{ij}=\;R_{w}=1.0$

**Table 1F:** **Input**.

**Type**	**Description**
Baseline Organization	Model three digits of the hand as non-overlapping 45 × 15 subarrays of 45 × 45 external input grid.
Baseline Trial	A 350 ms trial in which a contiguous **patch** of S layer units of fixed-size (7 × 7) is set to ON with magnitude (*v* = 1.0+ϵ(*t*)) for 50 ms following a 100 ms pre-stimulus period; in each time step during stimulus presentation the input is vector normalized to magnitude 4.0; patch position is random and respects digit “boundaries”
Baseline Cycle	One cycle (T) consists of the block of trials (N_b_) required to present every possible Baseline Trial once in random order.
Syndactyly Organization	Model surgical “fusion” of two digits as a 45 × 30 subarray of the 45 × 45 external input grid; the remaining 45 × 15 subarray serves as the “control” digit
Syndactyly Trial	For the control digit same as a Baseline Trial. For the fused digits same as Baseline Trial, but the input patch position ranges over the 45 × 30 subarray. Input patches delivered to both the control and fused digits respect the “boundary” between the control digit and fused digits.
Syndactyly Cycle	One cycle (T) consists of the block of trials (N_b_) required to present every possible Syndactyly Trial once in random order.
Receptive Field (RF) Probe Trial	A 350 ms trial in which a **single** S layer unit is set to ON with magnitude (*v* = 1.0+ϵ(*t*)) for 50 ms following a 100 ms pre-stimulus period.

**Table 1G:** **Measurements**.

**Type**	**Description**
Unit Activity	Membrane potential (*v_*i*_*), average firing rate (*r_*i*_*), connection weights (*w_*ij*_*)
Receptive Field	Receptive field is defined as the subset of layer S nodes that drive the given cell firing rate to greater than 50% of the maximum firing rate observed in that cell across the entire set of receptive field probe trials. Receptive field response magnitude is defined as the maximum observed ratio of mean stimulus-period firing rate to mean pre-stimulus period firing rate.
	Receptive field location is defined as the maximum likelihood estimate of position over the set of layer S nodes in the receptive field (e.g., sample estimate of the mean), where each layer S node is assigned a coordinate on a uniform 2D grid. Receptive field orientation is approximated using maximum likelihood estimation (e.g., sample estimates) of the covariance matrix of the positions of the layer S nodes in the receptive field.
	For display purposes, the mean and covariance values are used as input parameters to a unimodal Gaussian distribution to generate an estimate of the receptive field extent (e.g., 50% level). Note: with such a procedure multi-peaked or discontinuous receptive fields will display as single large receptive fields so it is important to inspect and to interpret map displays accordingly.

The model neuron is an RC-time constant membrane potential (*v*_*i*_) subject to depolarization and hyperpolarization through weighted input connections. The S layer input unit is, in addition, driven by an external ON/OFF stimulus. Average spiking rate (*r*_*i*_) for the neurons and input units are modeled by passing the corresponding membrane potential (*v*_*i*_) through a sigmoidal compression function. See Table 1D.

Synapses (connection weights) are initially assigned values from a uniform distribution. These are subsequently adapted according to exponential decay, a simple covariance rule, and normalization. Weight adaptation (plasticity) is controlled by an external ON or OFF signal. When ON, connection weight adaptation occurs at each time step △*t* in the numerical integration. See Table 1E. When ON, connection weights are normalized at the end of each stimulus presentation trial. See Table 1F.

### Model inputs and experimental protocol

The present study is organized as an “experimental” track consisting of three phases (baseline, syndactyly, and syndactyly release) and a corresponding “control” track (baseline, syndactyly control, and syndactyly release control). Each phase consists of a set of 15 cycles. Each cycle consists of a set of 0.350 s duration trials. Model inputs are presented and model outputs measured during each such trial. See Table 1F.

Phase I “baseline refinement” is the simulation that models the emergence and maintenance of somatotopy from random initial conditions. A single baseline refinement cycle consists of stimulating all possible patches of a fixed size one at a time per trial *while respecting digit “boundaries.”* The number of 7 × 7 patches in a 45 × 45 baseline network is 351 per digit (total 1053/cycle). The baseline refinement phase consists of 15 cycles (15,795 trials). Weight adaptation is ON during baseline refinement. A receptive field map is derived after each cycle. See Table 1G. During the receptive field mapping procedure weight adaptation is OFF.

Phase II “syndactyly refinement” is the simulation that models the response of a baseline refined network to simulated digital syndactyly. The driving stimuli are spatially localized, correlated inputs on layer S. A single syndactyly refinement cycle consists of stimulating all possible patches of a fixed size one at a time per trial *while respecting the D2-D3 boundary, but ignoring the D1-D2 boundary*. The number of 7 × 7 patches in a 45 × 45 syndactyly network is 351 for D3 and 936 for the syndactyly D1+2 (total 1287/cycle). The syndactyly refinement phase consists of 15 cycles (19,305 trials). Weight adaptation is ON during syndactyly cycles. A receptive field map is derived after each cycle. During the receptive field mapping procedure weight adaptation is OFF.

Phase III “syndactyly release” repeats Phase I, but uses the syndactyly network as its starting point. Weight adaptation is ON during syndactyly release. A receptive field map is derived after each cycle. During the receptive field mapping procedure weight adaptation is OFF.

The Experimental track evolves a single network of fixed size and fixed input patch size through Phase I (baseline refinement), Phase II (syndactyly) and Phase III (syndactyly release). On the Experimental track, digits 1 and 2 are the “experimental” digits; D3 provides within-network control.

The Control track starts with the Experimental Phase I baseline refined network and subjects it to two additional complete baseline refinement runs. In this protocol D3 undergoes the same total number of stimulation trials overall on both the Control and Experimental tracks. Similarly, all areas of digits 1 and 2 (sufficiently distant from the D1–D2 border) undergo the same number of stimulation trials.

**Figure 1:**
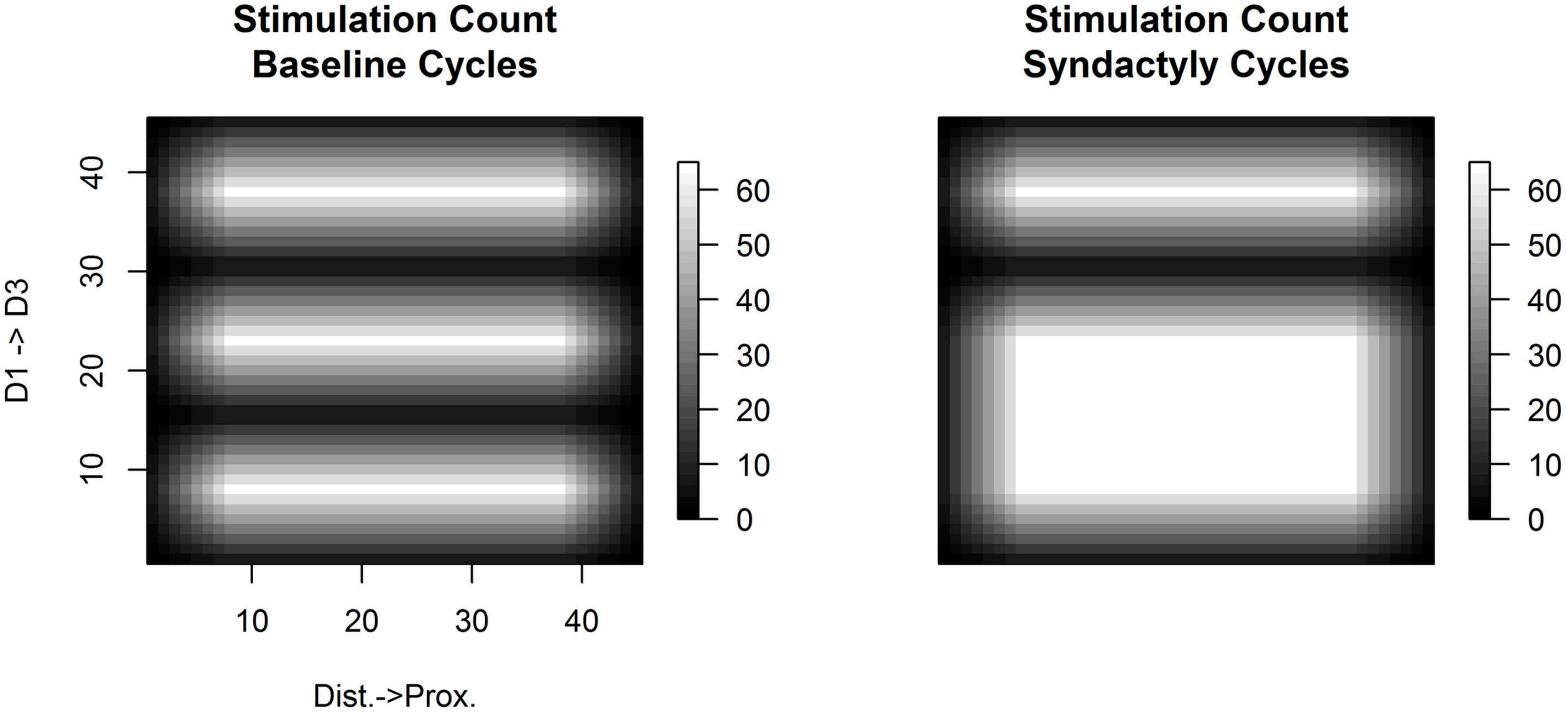
**Relative frequency of stimulation**. A single experimental “cycle” consists of the application of all possible stimuli of a given fixed size (e.g., 7 × 7) once in random sequence across the simulated three-digit hand. Shown are the spatial distributions of the number of times each input layer node is stimulated per cycle under normal **(left)** and syndactyly **(right)** configurations. There is a differential amount of stimulation within-digits (more along the medial longitudinal; relatively less toward the lateral edges), but the stimulation count profile is the same (per node) across digits. Across digits this experimental design controls for stimulation count effects in results observed in syndactyly zones (digits 1 and 2). (Values shown x- and y-axes are the node labels in the 45 × 45 network).

Figure 1 shows the per trial stimulation count for baseline and syndactyly trials normalized to the same magnitude scale for the network used throughout this study. This protocol controls for the effects of overall stimulation count.

### Model outputs

Simulated receptive field mapping is a two-step process. A Receptive Field Probe trial is run one at a time once for each layer S node (*N* = 2025) and the cortical layer E and I cell activity stored. See Table 1F. Next, the recorded data is post-processed to calculate receptive field response magnitude, area (extent), orientation and position, for each cortical layer E cell *and* I cell. See Table 1G.

## Results

### Temporal response during receptive field probe

**Figure 2:**
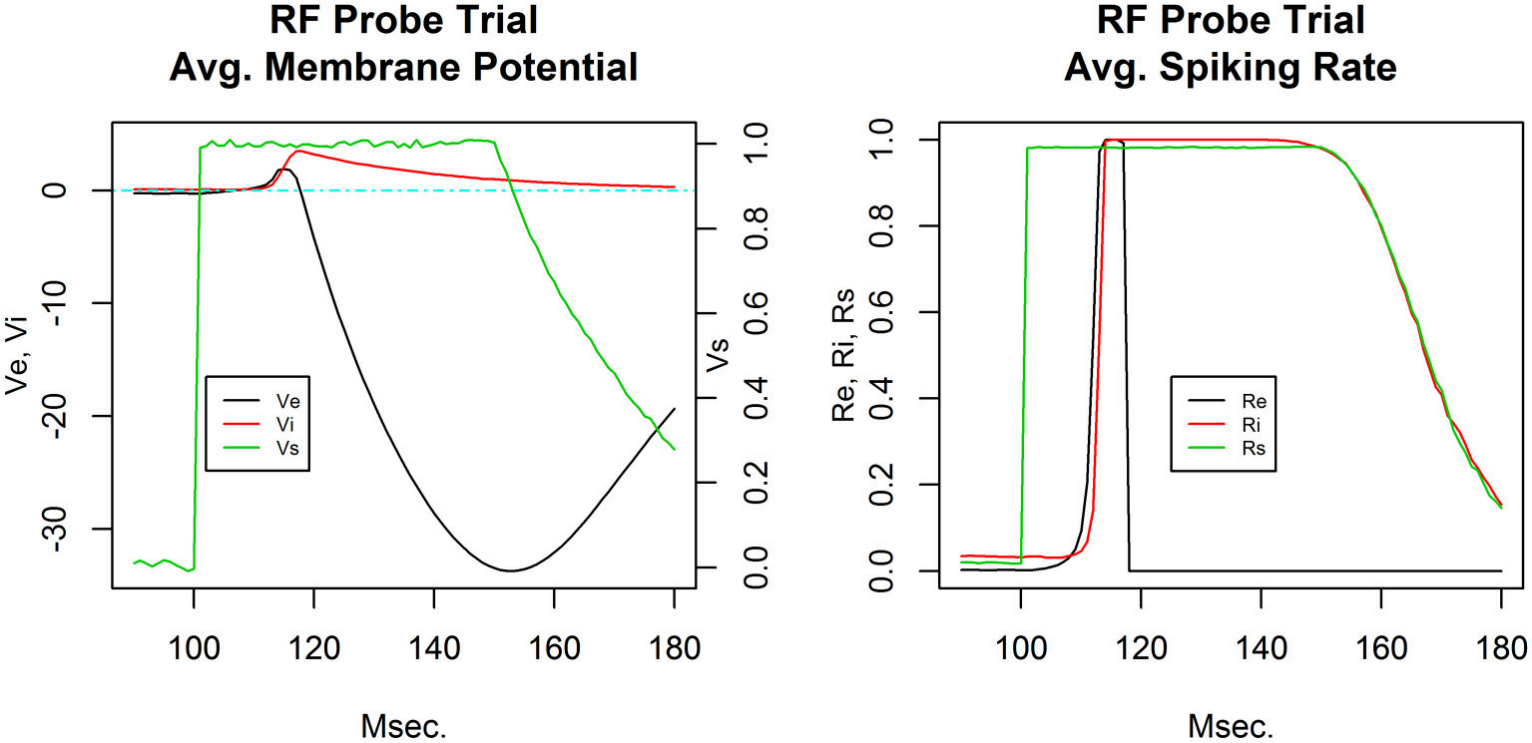
**Receptive field probe trial temporal response**. Average membrane potential **(left)** and action potential firing rate **(right)** for the excitatory cell (black) and inhibitory cell (red) in a single cortical column. The receptive field probe is modeled as a depolarization of the input layer node (green). For visual clarity, the input layer node depolarization is plotted with its own scale (shown on **right**) in the average membrane potential plot.

Figure 2 shows typical receptive field probe trial activity from a single column C layer E cell and I cell. The response consists of a low latency excitatory response in the E cell and a delayed strong excitatory response in the corresponding I cell. Both excitation levels build during the early portion of stimulus presentation. The E cell subsequently undergoes strong hyperpolarization. I cell activation decays with a dominant RC-time constant characteristic. The time series shown is from a network produced by the Phase I baseline refinement protocol.

### Excitatory receptive field centroids

**Figure 3:**
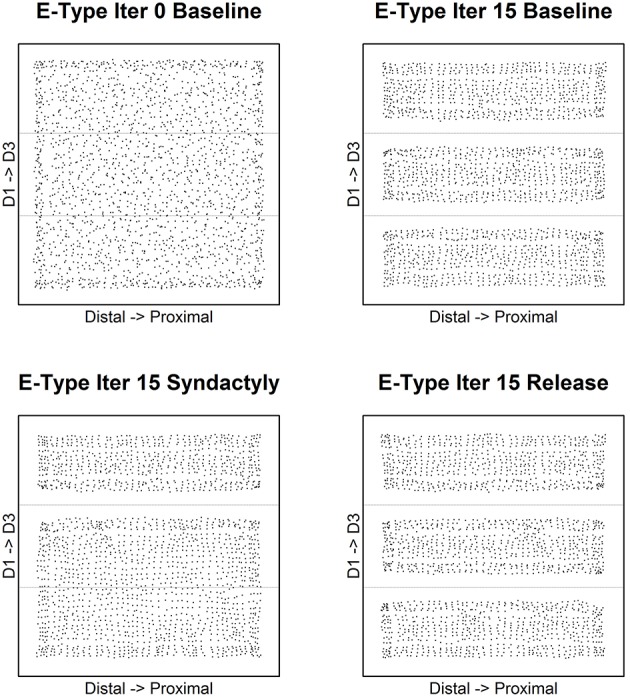
**Excitatory receptive field centroid position location**. Map of excitatory cell receptive field centroids demonstrates the emergence and reorganization of cortical somatotopy before, during and following digital syndactyly. Baseline iteration 0 **(top left)** is the network in its initial random condition. Excitatory cell somatotopy after 15 cycles of refinement is shown **(top right)**. The distributions of centroids following 15 syndactyly cycles **(bottom left)**. That the changes driven by syndactyly are reversible is shown by a restoration of normal somatotopy following a single iteration **(bottom right)** and 15 cycles of baseline refinement **(bottom right)**. The experimental design affords the opportunity to compare neural mechanisms that maintain the representational discontinuity between digits 2 and 3 throughout the experimental protocol with those whose initial representational discontinuity between digits 1 and 2 is established (baseline), obliterated (syndactyly) and then restored (release).

Figure 3 depicts the evolution of excitatory receptive field centroids over the course of the experimental track. The dashed lines in each panel indicate digit stimulation boundaries projected to topographically equivalent positions on the cortical lattice. Edge effects appear as expected and are limited to the extreme edges and corners of the network.

Of particular interest in this study are the boundaries between digits 1 and 2 (the experimental discontinuity) and between digits 2 and 3 (the control discontinuity). In the unrefined network the distribution of centroids is largely uniform and random. In the baseline refined network centroids cluster to either side of the digit midline. There are no centroids located on the nodes on either side of and immediately adjacent to digit boundaries (with the exception of a few nodes at network edge).

Following a single syndactyly refinement cycle the D1–D2 boundary is nearly obliterated. The border between digits 2 and 3 remains intact (with the exception of a few nodes at the network edges). Additional syndactyly refinement cycles fine-tune the centroid map.

Syndactyly release cycles reverse the effects of simulated syndactyly. Following a single cycle, a D1–D2 representational boundary begins to reemerge, though there remain centroids located on nodes immediately adjacent to the boundary. The D1–D2 boundary is fully restored with additional refinement cycles. The effects observed at the edges of the D2–D3 border during syndactyly are reversed as well. Additional syndactyly release cycles produce a map of excitatory receptive field centroids indistinguishable from a baseline refined map.

### Inhibitory receptive field centroids

**Figure 4:**
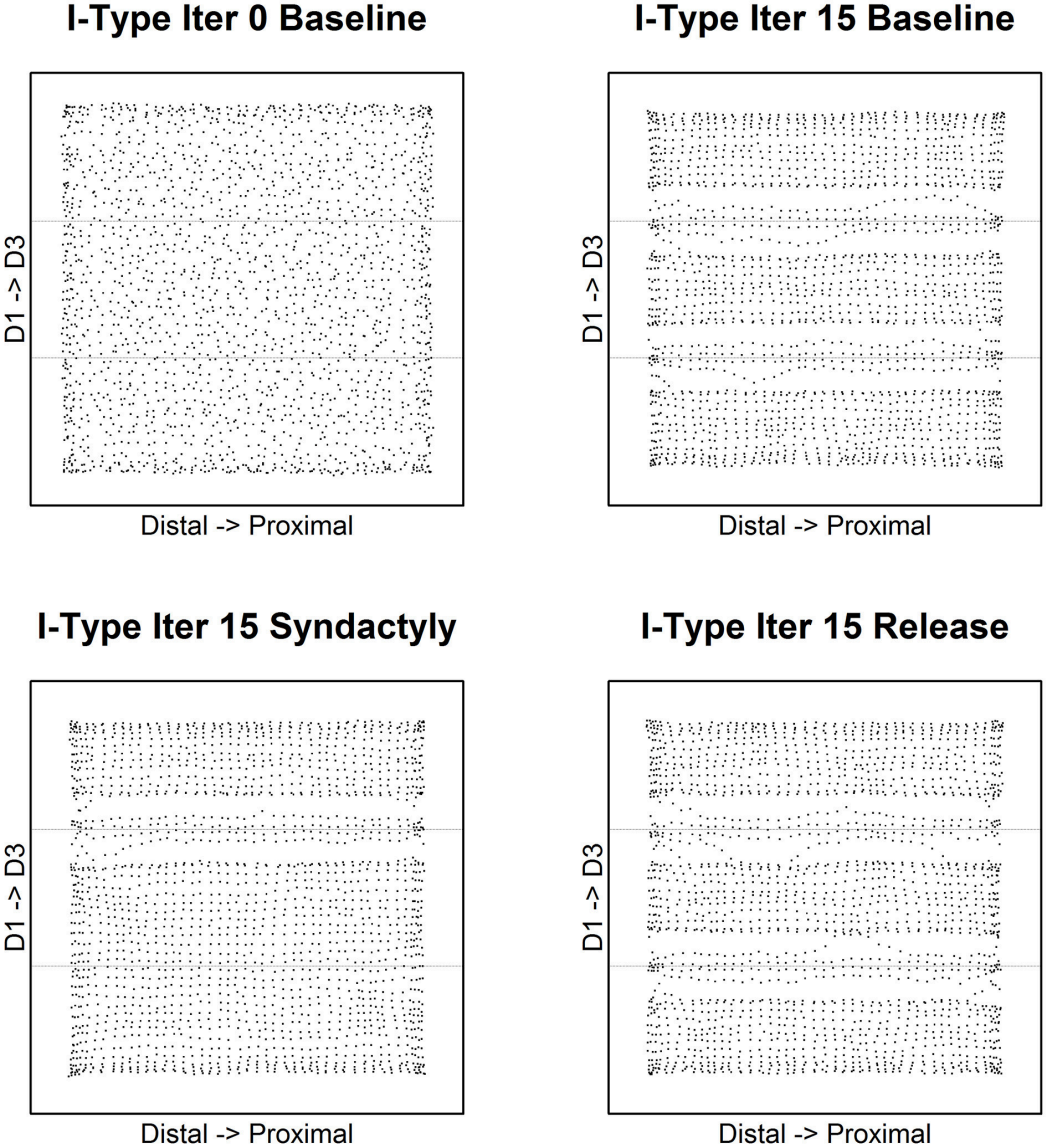
**Inhibitory receptive field centroid position location**. Map of inhibitory cell receptive field centroids demonstrates the emergence and reorganization of a strikingly different somatotopic organization compared with excitatory receptive fields before, during and following digital syndactyly. Baseline iteration 0 **(top left)** is the network in its initial random condition. Inhibitory cell type somatotopy after 15 cycles of refinement is shown **(top right)**. The distributions of centroids are shown following 15 syndactyly cycles **(bottom left)**. That the changes driven by syndactyly are reversible is shown by a restoration of the baseline inhibitory cell type somatotopy following 15 cycles of baseline-type refinement **(bottom right)**.

The results for inhibitory receptive field centroids are at once similar and strikingly different from those for excitatory receptive field centroids. Figure 4 illustrates the evolution of inhibitory receptive field centroids.

In the unrefined network the distribution of centroids is largely uniform and random. In the baseline refined network, in similar fashion to excitatory receptive fields, the inhibitory receptive field centroids cluster to either side of the digit midline. The E cell and I cell within these single cortical columns have coincident centroids.

A dramatic difference between excitatory and inhibitory receptive field centroid distributions is the observation of inhibitory receptive field centroids positioned on the nodes on either side of and immediately adjacent to digit boundaries. The E cell and I cell within these single cortical columns have divergent centroids.

Following a single syndactyly refinement cycle the D1–D2 boundary is nearly eliminated. The D2–D3 boundary remains intact. The consequence of additional syndactyly refinement cycles is a fine-tuning of the D1–D2 centroid map.

Syndactyly release cycles reverse the effects of simulated syndactyly. The border between D1–D2 begins to reemerge following a single baseline refinement cycle. Inhibitory receptive field clusters begin to migrate back toward their respective digit midlines and inhibitory receptive field centroids reappear located on the nodes on either side of and immediately adjacent to digit boundaries.

With additional syndactyly release cycles the D1–D2 boundary is fully restored. Inhibitory receptive field centroids reappear located on the nodes on either side of and immediately adjacent to D1–D2 boundary. At the D2–D3 boundary the inhibitory receptive field centroids located on the nodes on either side persist and undergo fine-tuning. The overall result is a map of inhibitory receptive field centroids nearly indistinguishable from a baseline refined map.

### Excitatory receptive field extents and overlap

**Figure 5:**
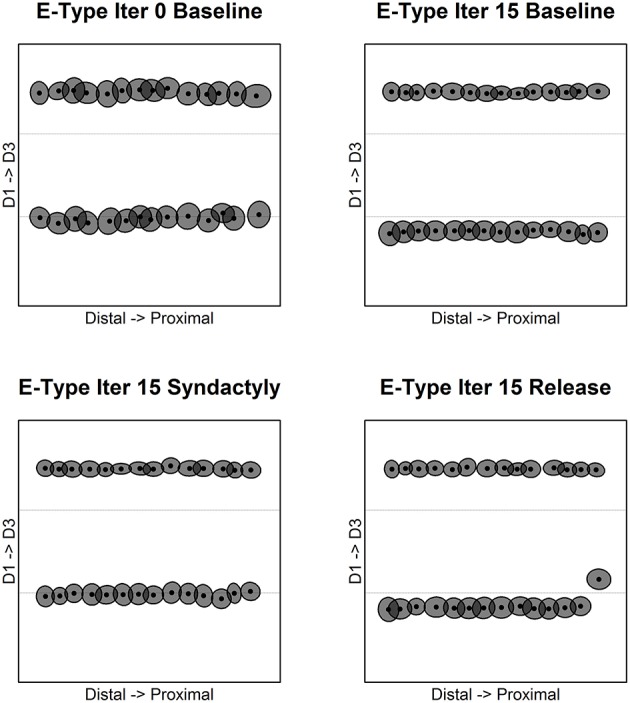
**Excitatory receptive field extents and overlap**. Excitatory cell receptive fields measured along a pair of representative longitudinal recording tracks in the simulated cortical layer conform to expectations of small, overlapping receptive fields that form smooth representational progressions. Without loss of generality only every third cortical column results are shown. In each panel the upper longitudinal track is along D3 midline (row 38 of 45 in the network; row 8 in the 15 row D3 representation); the lower track is along the longitudinal axis on the D1 side of the representational border between digits 1 and 2 (row 15 of 45 in the network; row 15 in the 15 row D1 representation). Baseline evolution is shown for iteration 0 **(top left)** and after 15 cycles of refinement **(top right)**. Receptive field extents and overlap are shown following 15 syndactyly cycles **(bottom left)**. That the changes driven by syndactyly are reversible is shown by a restoration of somatotopy following 15 cycles of baseline refinement **(bottom right)**.

Figure 5 depicts along representative fixed longitudinal recording tracks the evolution of excitatory receptive field extents and overlap. One recording track is located immediately adjacent to the D1–D2 border on the D1 side and serves as an “experimental” track. The second recording track is located along the midline of D3 and servers as a “control” track. Both tracks are selected on the basis that in the event of a strict one-to-one mapping of input layer to cortical layer these tracks would correspond to the zones indicated. The dashed lines in each panel indicate digit boundaries.

In the unrefined network, control track excitatory receptive fields are of similar though not uniform size, orientation and overlap. Experimental track excitatory receptive fields are of similar size, orientation and overlap. They are all double-digit D1, D2 in extent.

Baseline refinement yields features of normal somatotopy on both the experimental and control tracks. First, receptive fields are continuous, single-peaked, and roughly uniform in size (with possible though limited edge effects). Second, topographic order is preserved within digit representations. Third, receptive field overlap is greatest for immediately adjacent within-digit recording sites, and decreases monotonically with distance along the digit longitudinal axis. Last, representational discontinuities emerge. With the exception of a few sites at the extreme edges of the network, there are no longer any double-digit excitatory receptive fields on the experimental recording track. On the experimental track, receptive field centroids have shifted away from the D1–D2 border and toward the D1 midline, and receptive field sizes are smaller than those on the control track.

Following a single syndactyly refinement cycle the D1–D2 boundary is nearly obliterated. All of the excitatory receptive fields recorded on the experimental track have shifted their centroid away from the D1 midline and have acquired “double-digit” receptive field extent. Other features of normal somatotopy persist on both the experimental and control tracks. The consequence of additional syndactyly refinement cycles on the experimental track is a fine-tuning and greater uniformity of excitatory receptive field size. Differences in excitatory receptive field size between extreme edge and interior on the experimental track mitigate.

The effects of simulated syndactyly are reversible. Following a single cycle of baseline stimulation, excitatory receptive fields on the experimental track begin to migrate back in the direction of the D1 midline (not shown). Receptive field sizes, orientations and extent of overlap are more variable. Additional syndactyly release cycles result in a map of excitatory receptive field centroids indistinguishable from a baseline refined map.

### Inhibitory receptive field extents and overlap

The results for inhibitory receptive field extents and overlap are at once similar and strikingly different from those for excitatory receptive field extents and overlap.

**Figure 6:**
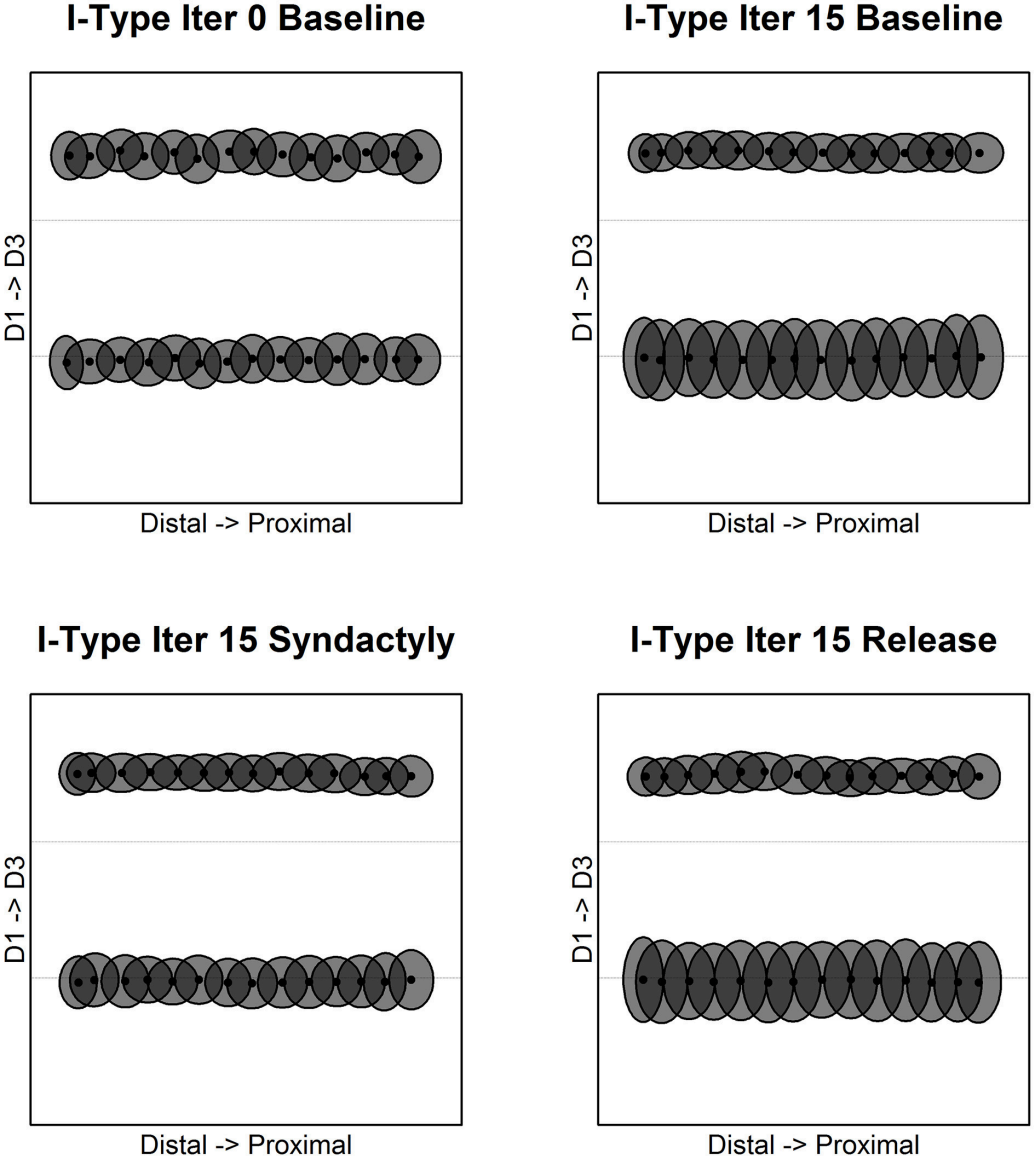
**Inhibitory receptive field extents and overlap**. Inhibitory cell receptive fields measured in the same nodes as in this figure show a strikingly different organization. In each panel the upper longitudinal track is along D3 midline (row 38 of 45 in the network; row 8 in the 15 row D3 representation); the lower track is along the longitudinal axis on the D1 side of the representational border between digits 1 and 2 (row 15 of 3450 in the network; row 15 in the 15 row D1 representation). Baseline evolution is shown for iteration 0 **(top left)** and after 15 cycles of refinement **(top right)**. Receptive field extents and overlap are shown following 15 syndactyly cycles **(bottom left)**.

Figure 6 depicts along fixed longitudinal recording tracks the evolution of inhibitory receptive field extents and overlap. The recording tracks are the same as in Figure 5.

In the unrefined network, control track inhibitory receptive fields are of similar though not uniform size, orientation and overlap. Experimental track inhibitory receptive fields are of similar size, orientation and overlap. Experimental track inhibitory receptive fields are double-digit in extent and larger than corresponding excitatory receptive fields.

Baseline refinement yields features of normal somatotopy on both the experimental and control tracks. First, receptive fields are continuous, single-peaked, and roughly uniform in size. Second, topographic order is preserved within digit representations. Last, receptive field overlap is greatest for immediately adjacent within-digit recording sites, and decreases monotonically with distance along the digit longitudinal axis.

There are differences between the control and experimental tracks. On the control track, inhibitory receptive field locations have not changed, but they are reduced in size and are single digit in extent. On the experimental track, inhibitory receptive field positions have not significantly translocated, but extent is much larger.

Comparison of the excitatory and inhibitory control tracks shows that inhibitory receptive field extent and overlap are greater than those of excitatory receptive fields in the same cortical column.

Comparison of the excitatory and inhibitory experimental tracks reveals profound differences in receptive field extent, orientation and degree of overlap. By visual inspection, inhibitory receptive field extent is much greater, there is less or no apparent orientation, and there is greater overlap. Whereas E cell receptive fields are single-digit in extent, I cell receptive field extents are double-digit and as will be shown below bimodal. A consequence of baseline refinement is a subset of cortical columns whose E cell and I cell receptive field characteristics profoundly differ. This divergence is located exclusively along (both sides of) representational discontinuities.

Following a single syndactyly refinement cycle changes are observed in both the control and experimental tracks. On the control track, by visual inspection the single cycle syndactyly inhibitory receptive field extents appear to be more variable, with some possible effects at the network edges, compared with the baseline refined results. It is likely that the changes observed on the control track are driven by a combination of network edge effects, frequency of stimulation and the profound changes occurring on the experimental track. On the experimental track, again by visual inspection, the inhibitory receptive fields are smaller and show relatively more orientation, compared with the baseline refined results. The consequence of additional syndactyly refinement cycles on the experimental track is a further reduction in inhibitory receptive field extent and increased orientation.

Following a single post-syndactyly release cycle of normal stimulation, inhibitory receptive fields on the experimental track resemble those of a baseline refined network. Additional syndactyly release cycles result in a map of inhibitory receptive field centroids largely indistinguishable from that of a baseline refined map. The exceptions are minor in the form of a few inhibitory receptive fields whose size or orientation appear somehow “in between” that of the syndactyly refined and baseline refined network.

**Figure 7:**
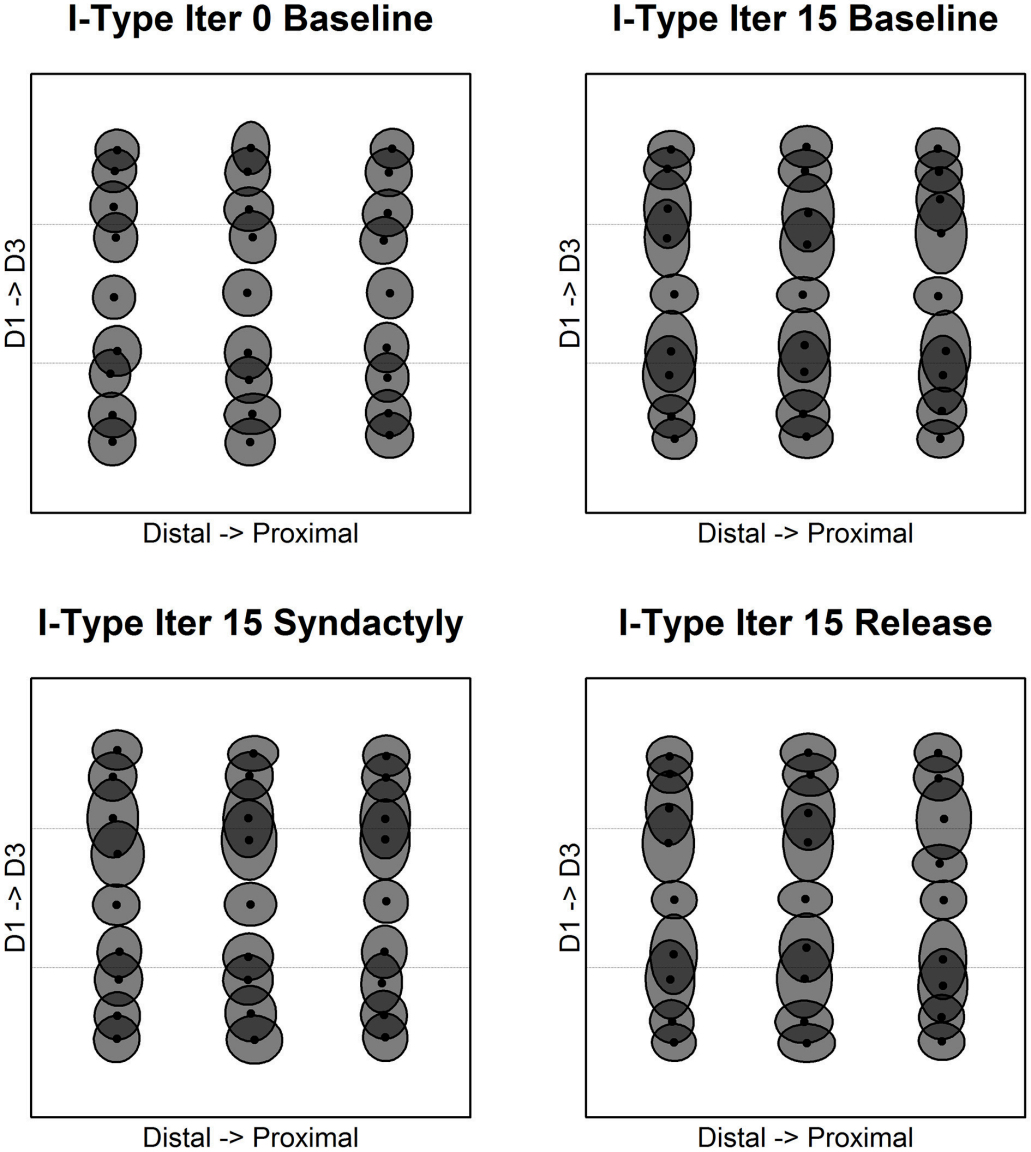
**Inhibitory receptive field extents and overlap**. Inhibitory cell receptive fields measured across digits along recording tracks fixed on the distal-proximal representational axis in the simulated cortical layer. Shown are representative columns “within representation” and those straddling representational borders. Each panel shows three tracks: midline of distal phalange **(left)**, midline of middle phalange **(middle)**; and midline of proximal phalange **(right)**. The progression of panels is same as Figure 6.

Figure 7 depicts along representative fixed cross-digit recording tracks the evolution of representative inhibitory receptive field extents and overlap. Each recording track is located in approximately the middle of the distal, mid, proximal phalange, respectively. Tracks are selected on the basis that were there a one-to-one mapping of input layer to cortical layer these tracks would correspond exactly to the zones indicated.

**Figure 8:**
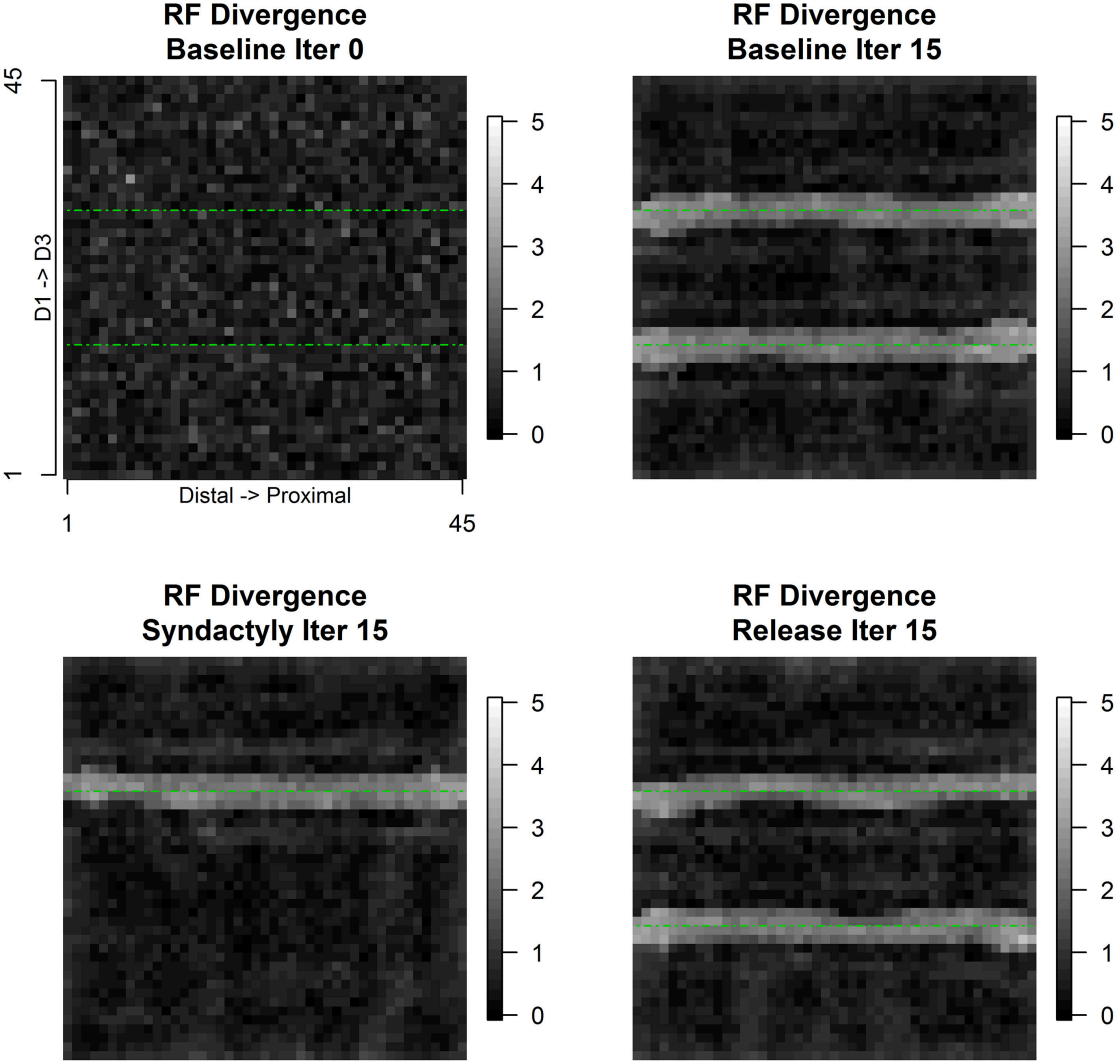
**Intracolumnar excitatory and inhibitory receptive field centroid divergence**. Receptive field centroid divergence is defined as the Euclidean distance on the input layer lattice between the excitatory receptive field centroid and the inhibitory receptive field centroid. Immediate neighbors on the input lattice differ by 1 unit horizontally or vertically and by 1.414 diagonally. Intracolumnar centroid distances greater than 2 diagonal units—divergence—are found exclusively straddling the digit representational borders. The progression of panels is same as Figure 6.

Figure 8 depicts the evolution of the spatial pattern of intracolumnar excitatory and inhibitory receptive field centroids divergence over the course of the experimental track. Divergence is defined as the Euclidean distance between the E and I receptive field centroids. Assuming unit distance separation between input layer units, a divergence value >1.414 represents a shift of one unit diagonally. Divergence values are highest in the bands of columns immediately adjacent to and on both sides of representational discontinuities. *These numerical results confirm the visual observation to be shown below that in the regions near discontinuities the E cells are unimodal, single-digit and small, and the I cells are bi-modal, double-digit and large.*

### Detailed analysis of individual simulated cortical columns

Detailed analysis of individual simulated cortical columns reveals characteristic differences between columns located within a cortical representational zone and those immediately adjacent to and on either side of a cortical representational discontinuity.

**Figure 9:**
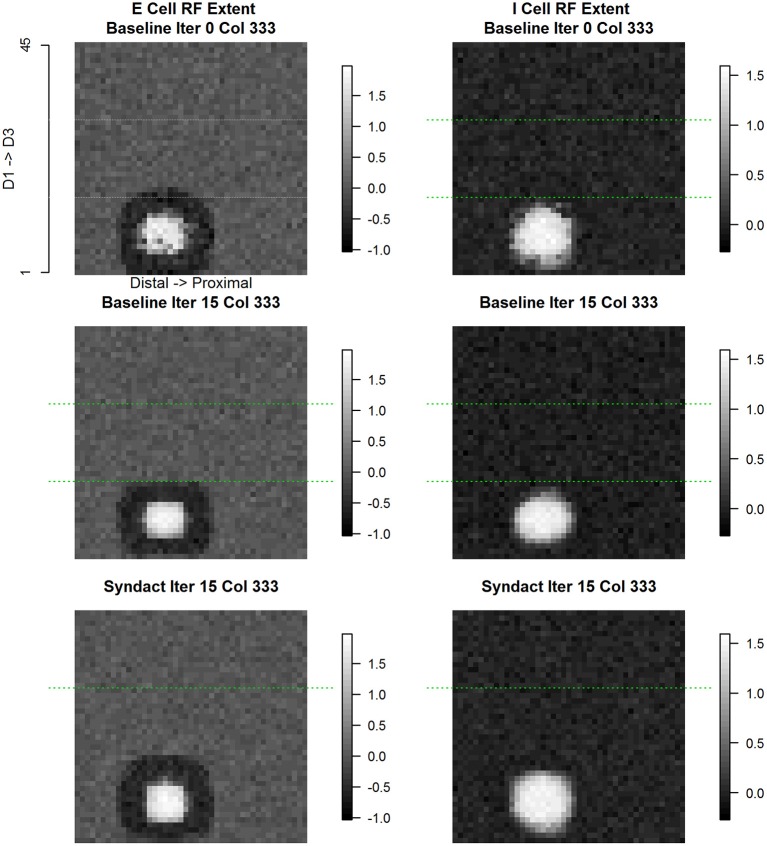
**Column #333 (within-representation) concentric same node E cell and I cell receptive field response**. The position of this column (#333) is adjacent to the D1 cortical representational midline. Each point in the spatial map represents the log (base 10) of the maximum response elicited in column #333 in response to a receptive field probe applied at the topologically equivalent nodes over the entire 45 × 45 input layer. Excitatory receptive field extent for initial random conditions **(top left)**, following 15 cycles of baseline refinement **(middle left)**, and following 15 cycles of syndactyly refinement **(bottom left)**. Inhibitory receptive field extent for initial random conditions **(top right)**, following 15 cycles of baseline refinement **(middle right)**, and following 15 cycles of syndactyly refinement **(bottom right)**. Typical of within-representational cortical columns, the excitatory and inhibitory receptive fields maintain a concentric, single-digit, and unimodal characteristic.

Column (#333) is within the D1 representation adjacent to the D1 representational midline. Figure 9 shows the evolution of E cell and I cell receptive field extent and response magnitude across experimental conditions. In both cases, what emerges from baseline refinement and persists through syndactyly refinement are continuous, coincident, single-digit, single-peaked receptive fields with common orientation. The inhibitory receptive field is larger in extent and lower response magnitude than the excitatory receptive field.

**Figure 10:**
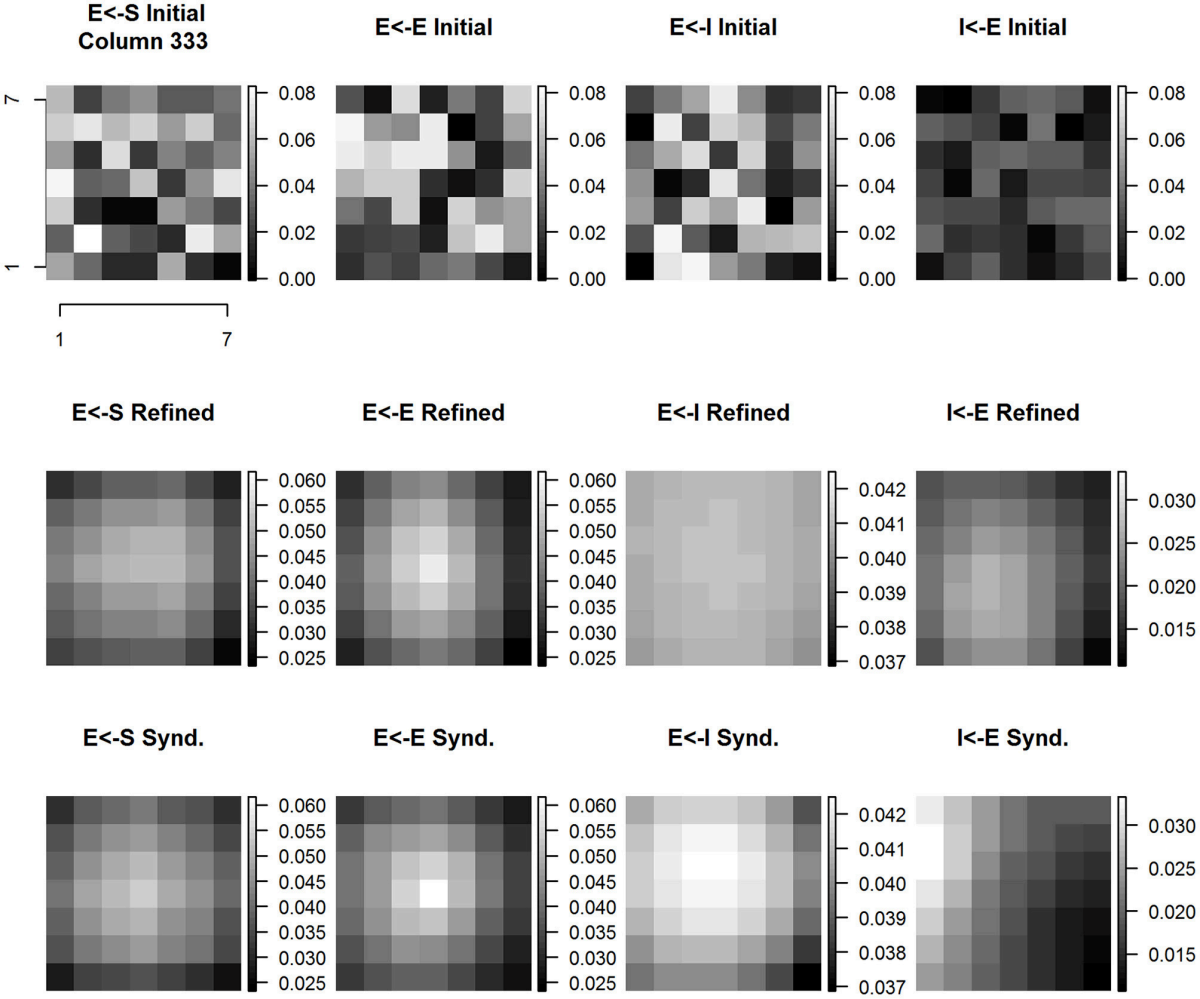
**Column #333 (within-representation) concentric same node E cell and I cell receptive field extent spatial distribution of input synaptic weights by type and experimental condition**. Each panel shows a (7 × 7) magnified view of the spatial pattern of input synaptic weights by type and experimental condition in the node whose E cell and I cell receptive field extent and position are shown in Figure 9. Three experimental conditions are shown: initial baseline conditions before refinement **(top row)**; following 15 baseline refinement cycles **(middle row)** and following 15 syndactyly refinement cycles **(bottom row)**. Four input synapse types are shown: input layer (S) to Excitatory (E) cell **(left-most column)**; local E cell to E cell **(second from left column)**; local Inhibitory (I) cell to E cell **(second from right column)**; and local E cell to I cell **(right-most column)**.

Figure 10 shows the evolution of E cell and I cell spatial patterns of incoming synaptic weights across experimental conditions. Each panel is a zoomed 7 × 7 image with its own magnitude scale to enhance contrast and to reveal fine spatial detail. The coordinates of column #333 are at center (4, 4) of each subplot. The interpretation of the data shown in each panel is that the cell positioned in the center receives inputs from itself and neighboring cortical columns. The anatomical parameters selected for this model establish the maximum number of input weights as coming from a 7 × 7 grid of cortical columns. Each panel shows all of the incoming weights to the cortical column located at the center. Time evolves within the figure columns from top to bottom.

The spatial pattern of input layer to excitatory cell synaptic weights following baseline refinement is a single-peaked, continuous distribution concentrated within a sub-zone slightly offset from center. The configuration remains stable throughout syndactyly refinement. The spatial pattern of excitatory to excitatory synaptic weights is single-peaked and remains stable from baseline refinement through syndactyly refinement. Notably, following baseline refinement, the spatial pattern of inhibitory to excitatory weights is nearly uniform with little or no orientation. The range of values of the weights is wider following syndactyly, though still narrow (ratio of maximum to minimum is 1.16 in contrast to excitatory to excitatory connection type maximum to minimum ratio of 2.7). The inhibitory cell in cortical column #333 receives weighted inputs from a 7 × 7 grid of excitatory cells. Following baseline refinement, the spatial pattern of weights is single-peaked, through broad, continuous, and slightly offset from center away from the discontinuity between digits 2 and 3, and slightly proximal. Following syndactyly refinement, the spatial pattern remains single-peaked, continuous, but is more concentrated in a zone distal to center and slightly toward the border between digits 2 and 3.

The above analysis was repeated on all applicable cortical columns with similar results. These results establish a profile of characteristic spatial patterns of incoming excitatory and inhibitory synaptic weights within cortical representational zones. The stage is set for a comparative analysis with cortical columns straddling a cortical representational discontinuity.

**Figure 11:**
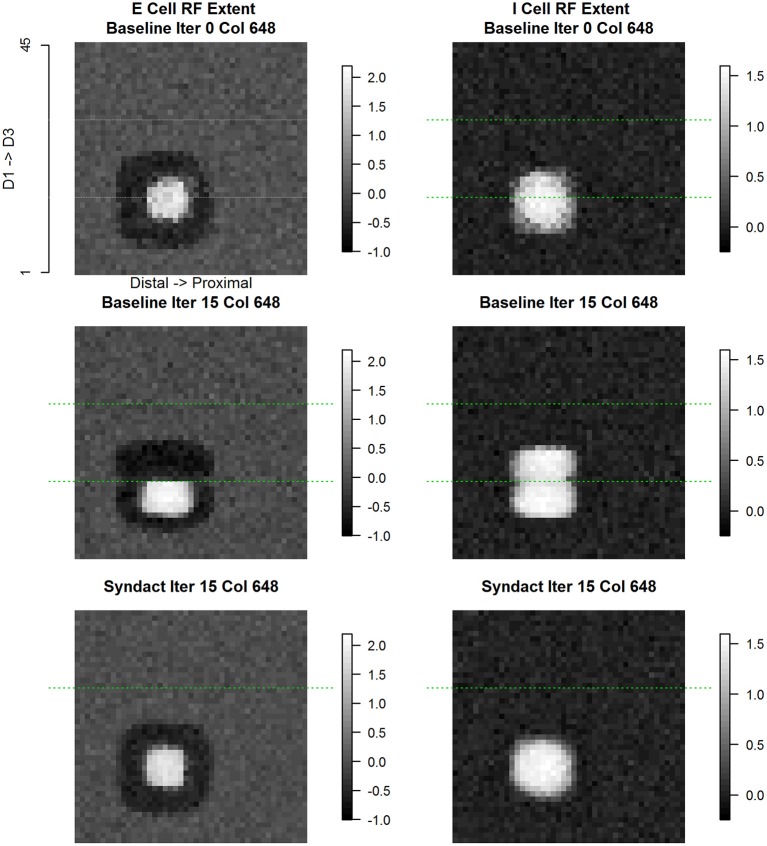
**Column #648 (representational boundary-adjacent) divergent same node E cell and I cell receptive field extent**. Same layout as Figure 9. Each point in the spatial map represents the log (base 10) of the maximum response elicited in column #648 in response to a receptive field probe applied at the topologically equivalent nodes over the entire 45 × 45 input layer. The position of this column (#648) is on the D1 side immediately adjacent to the D1–D2 representational boundary. Typical of representational boundary-adjacent cortical columns, the excitatory and inhibitory receptive fields have divergent characteristics in the baseline condition: excitatory receptive field extents are single-digit, limited extent, and unimodal; inhibitory receptive field extents are double-digit, large, and bimodal, where one of the modes is concentric with the excitatory receptive field. Under syndactyly, these cells' receptive field properties reorganize to those indistinguishable from within-representational cells and reacquire boundary-adjacent characteristics upon release from syndactyly.

Cortical column (#648) lies immediately adjacent to the D1-D2 cortical representational discontinuity. Figure 11 shows the evolution of E cell and I cell receptive field extent and response magnitude across experimental conditions. In the case of the excitatory cell, what emerges from baseline refinement and persists through syndactyly refinement is a continuous, coincident, single-digit, single-peaked receptive field. The excitatory receptive extent follows a characteristic evolution across experimental conditions: double-digit at initial random state; single-digit as a result of baseline refinement (including translocation of the receptive field centroid); and double-digit (including translocation of the receptive field centroid) as a result of syndactyly.

The inhibitory receptive field—from the same cortical column #648—undergoes a dramatically different evolution. The progression from initial conditions to a baseline refined map results in a large, double-digit receptive field. The progression from baseline refined map to a syndactyly refined map—wherein the D1-D2 representational boundary is obliterated—results in an inhibitory receptive field single-digit profile characteristic of a normal within-representational inhibitory cell. Not shown is the further result that following syndactyly release, the double-digit inhibitory receptive field characteristic is restored and is indistinguishable from that recorded at baseline refinement.

**Figure 12:**
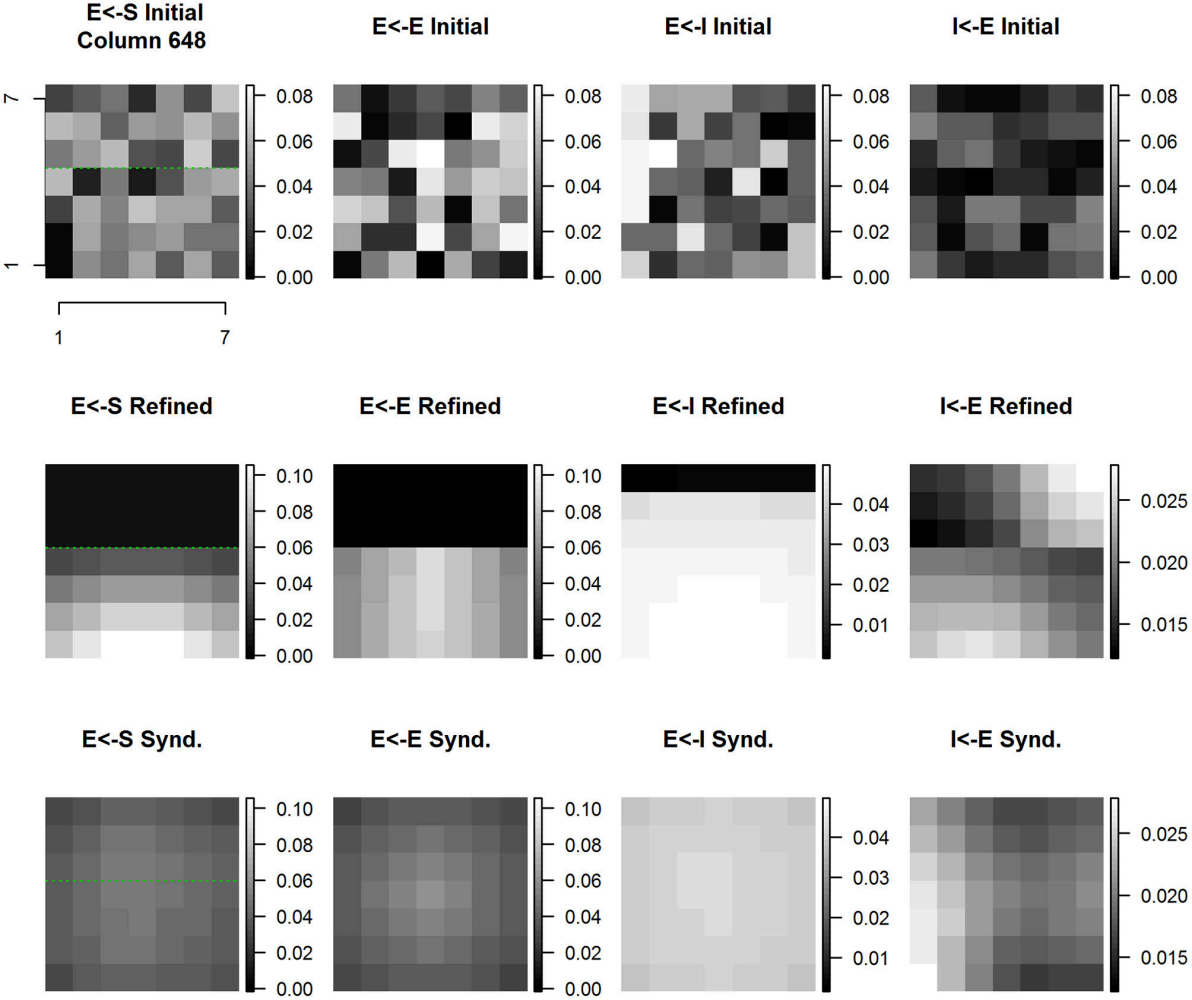
**Column #648 divergent same node E cell and I cell receptive field extent spatial distribution of input synaptic weights by type and experimental condition**. Each panel shows a magnified view of the spatial pattern of input synaptic weights by type and experimental condition in the node whose E cell and I cell receptive field extent and position are shown in Figure 11. Same layout as Figure 10.

Figure 12 shows the evolution of E cell and I cell spatial patterns of incoming synaptic weights across experimental conditions for cortical column #648. The organization of Figure 12 is the same as Figure 10. For every type of synapse studied there is a dramatic progression of changes across experimental conditions from initial random conditions to baseline refinement to syndactyly refinement.

The spatial pattern of input layer to excitatory cell synaptic weights undergoes shifts that are highlighted in comparison with the position of the D1-D2 border indicated by the green line. The result of baseline refinement is a tight cluster of weights exclusive to D1 locations spanning the region between the D1 midline and the D1-D2 representational boundary. As a result of syndactyly there is a significant shift such that there is a large, single-peaked continuous pattern spanning locations on both D1-D2. The resulting distribution is indistinguishable from that of a normal baseline within-representational zone profile. Not shown is the further result that following syndactyly release refinement the baseline refined map spatial pattern is largely, if not completely, recovered.

The changes in spatial pattern of excitatory to excitatory cell synaptic weights across experimental conditions mirrors those of the sensory afferent to excitatory cells. The result of baseline refinement is a broad nearly flat distribution of weights originating from a subset of connected excitatory cells whose receptive fields, in turn, are located exclusively on D1 locations spanning the region between the D1 midline and the D1-D2 boundary. The ratio of maximum to minimum connection weight within-digit (proximal-distal axis) is ~2x, whereas the ratio of maximum to minimum connection weight across digits is >100x (in favor of D1). As a result of syndactyly there is a significant shift such that there is a large, nearly symmetric continuous pattern with contributions from excitatory cells whose receptive fields are “double-digit” from both D1 and D2, and where the ratio of maximum to minimum connection strength is ~2.25x. The resulting distribution is indistinguishable from that of a normal baseline within-representational zone profile. Following syndactyly release the baseline refined map spatial pattern is reacquired.

The spatial pattern of inhibitory to excitatory cell synaptic weights changes shares many, but not all of the features of those described for sensory afferent to excitatory, and excitatory to excitatory. The notable difference is that as a consequence of baseline refinement the excitatory cells receive inputs from inhibitory cells on both sides of the representational border. The source of inhibitory inputs from D2 is restricted to the two rows wide band immediately adjacent to the D1-D2 representational boundary. The spatial pattern of connection strengths is highly oriented across-digits, but nearly uniform within-digit representation. The results are consistent with and reveal at the level of individual synaptic weight patterns the basis for the observed double-digit inhibitory receptive fields. Following syndactyly release refinement, the baseline refined map spatial pattern is reacquired.

The progression of changes across experimental conditions of the spatial pattern of excitatory to inhibitory cell synaptic weights is unique compared to those discussed above. The result of baseline refinement is a multi-peaked distribution of weights whose sources are from excitatory cells on both sides of the representational border. There is a concentration from excitatory cells whose own receptive fields lie on the opposite side of the representational border (on D2) and with a slight distal offset. A broader, less concentrated contribution arises from excitatory cells whose own receptive fields lie on the same side (D1) of the representational border. These are shown in Figure 12 second row, rightmost column.

The above analysis was repeated on all applicable cortical column pairs straddling a representational border with similar results. These results establish a profile of characteristic spatial patterns of incoming excitatory and inhibitory synaptic weights that contribute to cortical representational discontinuities.

### Control experiments

Experimental Track II yields confirmatory control data (not shown) wherein the network undergoes substantially similar or nearly identical counts of stimulation but absent the syndactyly procedure. Throughout all phases, cycles and trial, the network maintains expected features of topographic organization and receptive field characteristics. Visual observation of topographic order and convergence of the network were confirmed with numerical measures of topographic order (not shown) (Goodhill and Sejnowski, 1996).

A control network was stimulated as a variation on Baseline Refinement in which the input patches ranged smoothly over the entirety of the input layer. The frequency of stimulation followed a similar pattern to that shown in Figure 1. With the exception of the first few outer rings of the network every input layer node received the same maximum number of stimulations per cycle. The control experiment confirms the absence of representational discontinuities and the absence of spatial patterns of excitation and inhibition characteristic of regions adjacent and on both sides of representational discontinuities. Results are consistent with the conclusion that the observations reported in this study are unique to representational discontinuity.

Multiple control experiments were conducted to test the hypothesis that the results obtained in this study are merely an artifact due the input stimulation pattern. In particular, during Baseline Refinement, there is a descending gradient in stimulation count moving away from midline toward the digit border or network edge. See Figure 1 **left**. A 15 × 15 input pattern eliminated this gradient. With the exception of the proximal-most and distal-most edges of the network all input layer nodes received the same count of stimulations. Rerunning the entirety of this study using the 15 × 15 input patch yielded no obvious differences (by visual inspection and numerical measure of topographic order) from the results reported above. The study was repeated with additional parameter variations. These included: (a) a range of input patch sizes from 5 × 5 to 15 × 15; (b) additional cycles up to 20 to confirm convergence; (c) varying input stimulus duration from 50 to 250 ms; (d) input stimulus magnitude [1.0, 2.0, 4.0, 8.0]; (e) varying the size of the local extent of connections from 3 × 3 to 4 × 4, and 5 × 5; (f) repeated trials (*N* = 5) with 45 × 45 networks with independent random number generator seeds; and (g) repeated trials (*N* = 3) with 75 × 75 networks with independent random number generator seeds. The results reported above were confirmed across all of these parameter variations and network trials.

A final set of control experiments sought to characterize the width of the “band” of double-digit inhibitory cells straddling the digit representational discontinuities. In a network of size 30 × 30 the width of the band of double-digit inhibitory cells was confined to a single row on either side of the discontinuity for a total of two rows. In the present 45 × 45 case there are two rows of such cells on both sides of the discontinuity for a total of four rows. Across repeated trials with 45 × 45 and 75 × 75 networks, the first row immediately to either side of the discontinuity inhibitory cells uniformly took on the characteristic of double-digit, bi-modal, large receptive field extent. In the next unit distance row away from the discontinuity and on both sides, inhibitory cells clearly maintained a double-digit characteristic, but for cells on the D1 (D2) side the receptive field extent and response magnitude favored D1 (D2) sites. In all cases the divergent characteristic was restricted at most to the first two rows on either side of a representational discontinuity.

## Discussion

### Summary of results

Simulation studies with a computational model of area 3b demonstrate novel emergent properties of inhibitory cells located exclusively in zones immediately adjacent to and on either side of cortical representational discontinuities. The model consists of a lattice of cortical columns wherein a simple covariance synaptic plasticity rule is paired with competitive normalization to tune *all* of the network connection weights. These inhibitory cells' input spatial patterns of synaptic weights, and receptive field extent, orientation and overlap on the input layer differ profoundly from those observed in: (a) the paired excitatory cell in the same cortical column, though with characteristic overlap; and (b) excitatory and inhibitory cells' properties in cortical columns located at a distance from the representational discontinuity. The stabilization of and reversible reorganization of the properties of these inhibitory cells contributes to the plasticity of simulated cortical representational discontinuity observed before, during and following release from simulated digital syndactyly between two of the digits in a simplified three-digit model of the hand. These simulations suggest that: (a) local and long-range receptive field structure and its plasticity may reflect fundamentally emergent properties of cortical neural networks of the present type; (b) where computational network connectivity is assigned *a priori*, results may mask these emergent properties; and (c) the magnitude of excitatory and inhibitory receptive field structure plasticity in and around representational discontinuities *must* be of greater magnitude—and thus may be more readily accessible to measurement by the neurophysiologist perhaps through the analysis of laminar differences—compared to within-representational zones.

### Limitations and extensions

Limitations and available extensions of the present study are in relation to variation of system parameters, certain as yet unexplored configuration which themselves may prove fruitful, and network stability conditions.

Results are reproduced and confirmed in networks obtained by systematically varying some, but not all network parameters. Parameters systematically varied include network size (*N* = 15 to *N* = 45 in steps of 5; and *N* = 75), input patch size (1 × 1–15 × 15), synaptic plasticity adaptation rate, number of cycles per run, input patch stimulation duration and trial length, among others. Variables not systematically varied are those relating to the anatomical spread of input node cortical layer connections, and the extent of local connectivity amongst connection types intrinsic to the cortical layer. One reason to not prioritize such analysis is that even with the present value of 7 × 7 local connectivity grids, the excitatory-excitatory cell connections values, for example, are observed to concentrate in sub-regions with rapid fall off to the edges. A systematic analysis could vary the local neighborhood connectivity from less than 7 × 7 to an all-to-all connection motif. Other limitations in the present study that could potentially be material include the use of only a single fixed input patch size in a given experiment and the presentation of only a single stimulus per trial. More complex characterizations of area 3b receptive field structure have been obtained experimentally (Dicarlo et al., 1998; DiCarlo and Johnson, 2002) and explored in a subsequent computational model (Detorakis and Rougier, 2014). With regards to such analyses, the present results beg the question as to the spatial distribution of the different observed receptive field structures with respect to representational discontinuities.

An unexplored observation in the present study is why the emergent receptive field sizes in the excitatory and inhibitory cells take on their particular observed values? What are the relative contributions of factors such as connection weight adaptation step size, input-to-cortical afferent spread, extent of local connectivity, and input patch grid size? Receptive field sizes in the present study remained within 15% average size with respect to the baseline refined network. Unexplored in the present study is how to interpret such results with respect to the cortical and inverse cortical magnification rules. The cortical magnification rule appears to hold in that cortical magnification of input zones is roughly proportional to relative frequency of stimulation of that zone. However, with receptive field sizes held relatively constant (the primary mode of reorganization is translocation of receptive field centroids) this study does not contribute directly to the question of inverse cortical magnification.

Network stability is a consideration and by itself a potentially fruitful area for further exploration using networks of the present type (Grajski and Merzenich, 1990; Binas et al., 2014.) The conditions over which present results are reproduced are those wherein a balance is obtained between excitation and inhibition locally in the network. This is empirically determined by setting of individual connection-type resource (R) parameters to assure return to steady state following presentation of an input stimulation in each trial. While the present results are reproduced across a range of conditions, a number of pathological conditions (seizure-type activity, degenerate cortical maps) are observed under not too “distant” conditions. Such conditions include manipulating the connection weight adaptation rate, boosting or reducing the effectiveness of the excitatory-to-excitatory connections, boosting or reducing the effectiveness of excitatory-to-inhibitory, and inhibitory-to-excitatory R values, or in other cases, by introducing various combinations of connection-type silencing to simulate cortical lesion or stroke. Pathologies are observed in both temporal and spatial domains.

In a characteristic temporal pathology, the network does not return to steady state following an input stimulus, but instead spiking oscillates in the range 20–40 Hz with amplitude at-the-limits. In certain of these cases, such as a preliminary cortical lesion experiment, oscillations were reduced and eventually eliminated following a conclusion of the standard 25-cycle refinement procedure. In this particular case, somatotopy was maintained (though with reduced cortical magnification as the same input layer was driving a smaller cortical network.) In other cases, whether temporal pathologies are observed, or not, the topographic map becomes “degenerate”: three to four representational zones emerge with exceptionally high cortical magnification of a few restricted zones on the input layer, and at times including double-digit receptive fields (even where the input stimulation patterns were single-digit only).

Finally, as with much of cortical neural network modeling, correspondence is most easily drawn with a salient feature of cortical neuroanatomy—the cortical column. While it is possible to observe complex and meaningful spatial-temporal dynamics in these lumped cortical column models, the present results do not explicitly take into account feed-forward and feed-back mechanisms involving sub-cortical contributions (Jones, 2000; Haider et al., 2013; Qi et al., 2014).

### Relationship to other studies

The contribution of the present study is both confirmatory and distinctly novel with respect to neurophysiological and computational studies of cortical columns and cortical neural networks.

#### Neurophysiology

Allard et al. (1991) demonstrated that temporally correlated afferent input activity plays a role in the emergence and plasticity of receptive fields and representational maps in the primary somatosensory cortex of adult monkeys. These findings are reproduced here through simulated differential use of digits in a model hand. When adjacent input layer patches on opposing digits were driven by temporally correlated stimulation (a direct consequence of simulated digital syndactyly) cortical receptive fields transformed to extend across the line of syndactyly onto the “joined” input layer patches of both fused digits and abolished the digit representational boundary. The model mechanism through which differential use transformed receptive field characteristics and representational topography was the operation of a covariance learning rule on excitatory and inhibitory synapses. The present model provides a framework within which to generate hypotheses to link neurophysiological observations and mechanisms with features of cortical neuroanatomy principally the cortical column.

Initial neurophysiological descriptions of somatosensory cortical columns remarked upon the similarity of receptive field size and overlap (Mountcastle, 1957, 1997). Subsequent studies observed that receptive field extent was smallest in layer IV, with overlapping similar receptive field sizes, or larger to substantially larger receptive field extent in supragranular and infragranular areas (Sur et al., 1985; Chapin, 1986; Haupt et al., 2004). Studies in rat whisker-barrel cortex have demonstrated short latency excitatory response in inhibitory cells (Brumberg et al., 1999; Petersen and Diamond, 2000) and inhibition of excitatory cells (Simons and Carvell, 1989; Brumberg et al., 1996) arising from activation of principal and adjacent whiskers. Studies of *in vitro* adult rat neocortical functional borders have described diverse excitatory and inhibitory outcomes following tetanic stimulation (Hickmott and Merzenich, 2002; Paullus and Hickmott, 2011).

Neuroanatomical contributions to laminar similarities and differences have been attributed to the laminar distribution of cell types and differential input-output patterns. Electrophysiological contributions have been attributed to the interplay between thalamo-cortically driven feed-forward excitation and feed-forward inhibition, recurrent intra- and inter-columnar excitatory and inhibitory circuits, and complex polysynaptic cortico-cortical connections. A possible functional contribution or consequence of receptive field size similarities and differences may reflect differential contributions to general principles of cortical layer IV processing. Miller et al. (2001), noted common organizing principles in a comparative analysis of cat primary visual cortex (V1) and the whisker-barrel fields of rodent primary somatosensory cortex with respect to response sharpening of excitatory ells and enhancement of response to effective stimuli. An additional possible role has been discussed with respect to modulating excitability and preventing hyperexcitability (Sun et al., 2006; Benali et al., 2008).

The present results are unique in generating novel hypotheses for neurophysiological testing. The results focus on observable laminar differences in receptive field size in neurophysiological recording tracks oriented perpendicular to the pial surface (e.g., cortical columns) in adult monkey area 3b and rat SI cortex. The experimental protocol might proceed to implement a standard receptive field mapping protocol to analyze adjacent cortical representations that each have sufficiently high cortical magnification and sufficiently small layer IV excitatory receptive field sizes. Such conditions may be met in the cortical representation of the glabrous skin surface of the phalanges of adjacent fingers of the hand in the monkey, or at the face-forearm representation in the rat. By sufficiently high cortical magnification is meant a representation that is large enough to support identification of a “within-representational” zone distant from all representational discontinuities. By sufficiently extensive representational borders is meant a region of discontinuity that permits multiple penetrations along and on both sides of the discontinuity. And by sufficiently small within-representational layer IV excitatory receptive field sizes is meant that increases in excitatory (inhibitory) receptive field size within- and between recording penetrations should be readily and obviously detectable using standard receptive field mapping procedures.

The present model predicts that with the above protocol the data should, in their simplest form, yield two characteristic, statistically significantly different, laminar difference profiles. One profile will correspond to the “within-representation” type (e.g., type WR) and the other will be the “boundary-adjacent” type (e.g., type BA). Furthermore, subject to experimental manipulation of the type described in this study, the experimenter will be able to cause reversible change from type BA to type WR, and from type WR to type BA. A variation on the above hypothesis is to induce plasticity in a representational discontinuity contemporaneously with single-unit or multi-unit recordings of both excitatory and inhibitory cells and taking into account laminar position.

#### Computational studies

The present computational model shares features in common with the literature of distributed self-organizing neural networks (Wilson and Cowan, 1972, 1973; von der Malsburg, 1973; Freeman, 1975; Willshaw and von der Malsburg, 1976; Takeuchi and Amari, 1979; Kohonen, 1982; Oja, 1982; Grajski and Merzenich, 1990; Joublin et al., 1996; Eglen and Gjorgjieva, 2009; Detorakis and Rougier, 2012, 2014; Harris and Mrsic-Flogel, 2013) which may be considered as a subclass (winner-take-all networks) of general models of unsupervised learning networks (Binas et al., 2014). The robustness of the results reported above in response to variations of the particular covariance rule was confirmed by varying connection strength relaxation time constant (α_*w*_), the weight adaptation step size (β_*w*_), the weight adaptation step size relaxation time constant (α_β_), and normalization frequency (i.e., per time-step vs per trial). Anecdotally, the formation of topographic maps with representational discontinuities was most sensitive to the weight adaptation step size (β_*w*_). Excitatory receptive field organization was more sensitive than inhibitory receptive field organization. For example, with much larger values than those listed in Table 1E (e.g., >10x), while inhibitory receptive field centroids distributed as reported, excitatory receptive field centroids organized in a degenerate fashion (see Section Limitations and extensions). The dependence of reported results on additional variations of the simple covariance rule (e.g., connection weight constraints other than normalization) or more complex dependencies (e.g., spike timing-dependence, Vogels et al., 2013; Kleberg et al., 2014) has not been explored.

The present computational model differs from and extends previous results in several ways. First, afferent and lateral network connectivity details are not assigned *a priori*. In prior studies it is not unusual to find that afferent connection strengths are modeled *a priori* as arising from a localized Gaussian distribution. This is distinct from assignment of spatial filtering of input so as to model skin effects. In the present case, afferent connection strengths are drawn from a uniform distribution. With respect to lateral connectivity, prior studies may define a parameterized lateral connectivity pattern in order to effect lateral inhibition. The present study initializes local lateral connection strengths from a uniform distribution. Second, network dynamics are simply those that evolve as given by the network equations with no additional higher order operations to select maximum responses or otherwise affect the time evolution of the network. Third, a quite simple covariance rule is applied to drive synaptic plasticity on **all** connections of the network, in particular inhibitory plasticity. In prior studies it is not unusual that only certain classes of connections are plastic and subject to a range from simple to complex adaptation rules. Fourth, detailed analysis of somatotopy, receptive field size and cortical magnification are provided separately for the component excitatory and inhibitory cell populations. Fifth, the experimental simulation protocol is designed to isolate a specific experimentally observed phenomenon (cortical representational discontinuity and its reorganization) so that its neural mechanism can be compared and contrasted with controls at the level of the evolution of spatial patterns by type of individual synaptic connections strengths. Finally, the present study suggests that perhaps there are additional features of neural networks that may be understood as fundamentally emergent.

### Future directions

This author is aware of few studies, until quite recently, comparable with the present study in which there is synaptic plasticity on all connections and, in particular, inhibitory synaptic plasticity (Grajski and Merzenich, 1990; Vogels et al., 2013; Binas et al., 2014; Kleberg et al., 2014). Present results establish a foundation for further numerical studies. How does the hand representation reorganize in response to targeted “silencing” or “amplification” of combinations of local cortical population subtypes? How does reorganization compare between within-representation and boundary-adjacent representational zones? Can the present model framework be extended to better relate features of cortical maps and their reorganization to sensation and the recovery of sensation in the clinical neurology of the hand?

The modeling study reported here may have potential application in personalized medicine analogous to that described for The Virtual Brain project (Leon et al., 2013; Falcon et al., 2016). Specifically, primary somatosensory cortical plasticity following release from syndactyly has been observed in adult humans (Mogilner et al., 1993; Stavrinou et al., 2007). Stavrinou et al., observed correlates of reorganization over the course of a roughly 6 h long preparation during which artificial syndactyly was imposed and released. Mogilner et al., observed reorganization of primary somatosensory cortical hand representation in two patients following surgical separation of webbed fingers. The degree of reorganization correlated with the severity of syndactyly. For one patient, reorganization yielded somatotopy and nearly normal representational area. For the second patient, though reorganization yielded distinct cortical locations for digits, the representational area was smaller than normal and nonsomatotopic. Could the present modeling framework, simulation protocol and measurements combined with individualized clinical data be combined to predict post-surgical cortical reorganizational outcomes? Could these predicted outcomes, in turn, be applied to predict extent and progression of recovery of sensation in the hand? Several extensions to the present hand model could potentially lead to clinically meaningful results. First, select one or more clinical assessments or psychophysical tasks (localization, two-point discrimination, vibratory sense) as behavioral touchstones (Dellon, 1981). Second, select a mechanoreceptor class or classes and skin surface model corresponding to the selected behavioral task (Zimmerman et al., 2014). Last, tailor model input layer, output layer and input stimulation pattern parameters through the translation of individual area 3b finger representation neuroanatomical and functional details (van Westen et al., 2004). Combined with the experimental protocol described in this paper, these extensions could generate model cortical reorganizational correlates of behavioral task performance under syndactyly, release from syndactyly, and potentially to other hand-related peripheral and central injuries.

## Software and data

Author-generated research (e.g., not for commercial use) C++ simulation code and R analysis scripts used to generate the Figures in this paper have been placed in a *GitHub* repository: https://github.com/kgrajski/KamilGrajski-Somatotopic-Discontinuity-Plasticity/.

## Author contributions

The author confirms being the sole contributor of this work and approved it for publication.

## Funding

This work was entirely self-funded.

### Conflict of interest statement

The authors declare that the research was conducted in the absence of any commercial or financial relationships that could be construed as a potential conflict of interest.

## References

[B1] Allard T. Clark S. A. Jenkins W. M. Merzenich M. M. (1991). Reorganization of somatosensory area 3b representations in adult owl monkeys after digital syndactyly. J. Neurophys. 66, 1048–1058. 1753275 10.1152/jn.1991.66.3.1048

[B2] Benali A. Weiler E. Benali Y. Dinse H. R. Eysel U. T. (2008). Excitation and inhibition jointly regulate cortical reorganization in adult rats. J. Neurosci. 28, 12284–12293. 10.1523/JNEUROSCI.1952-08.2008 19020022 PMC6671719

[B3] Binas J. Ruthishauser U. Indiveri G. Pfeiffer M. (2014). Learning and stabilization of winner-take-all dynamics through interacting excitatory and inhibitory plasticity. Front. Comput. Neurosci. 8:68. 10.3389/fncom.2014.00068 25071538 PMC4086298

[B4] Brumberg J. C. Pinto D. J. Simons D. J. (1996). Spatial gradients and inhibitory summation in the rat whsker barrel system. J. Neurophysiol. 76, 130–140. 8836214 10.1152/jn.1996.76.1.130

[B5] Brumberg J. C. Pinto D. J. Simons D. J. (1999). Cortical columnar processing in the rat whisker-to-barrel system. J. Neurophysiol. 82, 1808–1817. 10515970 10.1152/jn.1999.82.4.1808

[B6] Chapin J. K. (1986). Laminar differences in sizes, shapes and response profiles of cutaneous receptive fields in the rat SI cortex. Exp. Brain Res. 62, 549–5559. 3720884 10.1007/BF00236033

[B7] Dellon A. L. (1981). Evaluation of Sensibility and Re-education of Sensation in the Hand. Baltimore: Williams & Wilkins.

[B8] Detorakis G. I. Rougier N. P. (2012). A neural field model of the somatosensory cortex: formation, maintenance and reorganization or ordered topographic maps. PLoS ONE 7:e40257. 10.1371/journal.pone.0040257 22808127 PMC3395710

[B9] Detorakis G. I. Rougier N. P. (2014). Structure of receptive fields in a computational model of area 3b of primary sensory cortex. Front. Comput. Neurosci. 8:76. 10.3389/fncom.2014.00076 25120461 PMC4112916

[B10] DiCarlo J. J. Johnson K. O. (2002). Receptive field structure in cortical area 3b of the alert monkey. Behav. Brain Res. 135, 167–178. 12356447 10.1016/s0166-4328(02)00162-6

[B11] Dicarlo J. J. Johnson K. O. Hsiao S. S. (1998). Structure of receptive fields in area 3b of primary somatosensory cortex in the alert monkey. J. Neurosci. 18, 2626–2645. 9502821 10.1523/JNEUROSCI.18-07-02626.1998 PMC6793113

[B12] Eglen S. J. Gjorgjieva J. (2009). Self-organization in the developing nervous system: theoretical models. HFSP J. 3, 176–185. 10.2976/1.3079539 19639040 PMC2714953

[B13] Falcon M. I. Jirsa V. Solodkin A. (2016). A new neuroinformatics approach to personalized medicine in neurology: the Virtual Brain. Curr. Opin. Neurol. 29, 429–436. 10.1097/WCO.0000000000000344 27224088 PMC5536184

[B14] Freeman W. J. (1975). Mass Action in the Nervous Sytem. Cambridge, MA: Academic Press.

[B15] Goodhill G. J. Sejnowski T. J. (1996). Quantifying neighbourhood preservation in topographic mappings,”in Procceeding of 3rd Joint Symposium Neurol Comp. Vol. 6 (La Jolla, CA), 61–82.

[B16] Grajski K. A. Merzenich M. (1990). Hebb-type dynamics is sufficient to account for the inverse magnification rule in cortical somatotopy. Neural. Comput. 2, 71–84.

[B17] Haider B. Hausser M. Carandini M. (2013). Inhibition dominates sensory responses in the awake cortex. Nature 493, 97–102. 10.1038/nature11665 23172139 PMC3537822

[B18] Harris K. D. Mrsic-Flogel T. D. (2013). Cortical connectivity and sensory coding. Nature 503, 51–58. 10.1038/nature12654 24201278

[B19] Haupt S. S. Spengler F. Husemann R. Dinse H. R. (2004). Receptive field scatter, topography and map variability in different layers of the hindpaw representation of rat somatosensory cortex. Exp. Brain Res. 155, 485–499. 10.1007/s00221-003-1755-3 14745463

[B20] Hickmott P. W. Merzenich M. M. (2002). Local circuit properties underlying cortical reorganization. J. Neurophysiol. 88, 1288–1301. 10.1152/jn.00994.2001 12205150

[B21] Jones E. G. (2000). Cortical and subcortical contributions to activity-dependent plasticity in primate somatosensory cortex. Annu. Rev. Neurosci. 23, 1–57. 10.1146/annurev.neuro.23.1.1 10845057

[B22] Joublin F. Spengler F. Wacquant S. Dinse H. R. (1996). A columnar model of somatosensory reorganizational plasticity based on Hebbian and non-Hebbian learning rules. Biol. Cybern. 74, 275–86. 8867473 10.1007/BF00652228

[B23] Kaas J. H. Nelson R. Sur M. Lin C. S. Merzenich M. M. (1979). Multiple representations of the body within the primary somatosensory cortex of primates. Science 204, 521–523. 107591 10.1126/science.107591

[B24] Kleberg F. I. Fukai T. Gilson M. (2014). Excitatory and inhibitory STDP jointly tune feedforward neural circuits to selectively propagate correlated spiking activity. Front. Comput. Neurosci. 8:53. 10.3389/fncom.2014.00053 24847242 PMC4019846

[B25] Kohonen T. (1982). Self-organized formation of topographically correct feature maps. Biol. Cybern. 43, 59–69.

[B26] Leon P. S. Knock S. A. Woodman M. M. Domide L. Mersmann J. McIntosh A. R.. (2013). The Virtual Brain: a simulator of primate brain network dynamics. Front. Neuroinform. 7:00010. 10.3389/fninf.2013.00010 23781198 PMC3678125

[B27] Merzenich M. M. Kaas J. H. Sur M. Lin C. S. (1978). Double representation of the body surface within cytoarchitectonic areas 3b and 1 in S1 in the owl monkey (*Aotus trivirgatus*). J. Comp. Neurol. 181, 41–73. 98537 10.1002/cne.901810104

[B28] Merzenich M. M. Nelson R. J. Kaas J. H. Stryker M. P. Jenkins W. M. Zook J. M.. (1987). Variability in hand surface representations in areas 3b and 1 in adult owl and squirrel monkeys. J. Comp. Neurol. 258, 281–296. 3584541 10.1002/cne.902580208

[B29] Merzenich M. Recanzone G. H. Jenkins W. M. Grajski K. A. (1990). Adaptive mechanisms in cortical networks underlying cortical contributions to learning and nondeclarative memory. Cold Spring Harb. Symp. Quant. Biol. 55, 873–87. 2132864 10.1101/sqb.1990.055.01.082

[B30] Miller K. D. Pinto D. J. Simons D. J. (2001). Processing in layer 4 of the neocortical circuit: new insights from visual and somatosensory cortex. Curr. Opin. Neurobiol. 11, 488–497. 11502397 10.1016/s0959-4388(00)00239-7

[B31] Mogilner A. Grossman J. A. Ribary U. Joliot M. Volkmann J. Rapaport D.. (1993). Somatosensory cortical plasticity in adult humans revealed by magnetoencephalography. Proc. Natl. Acad. Sci. U.S.A. 90, 3593–3597. 8386377 10.1073/pnas.90.8.3593 PMC46347

[B32] Mountcastle V. B. (1957). Modality and topographic properties of single neurons of cat's somatic sensory cortex. J. Neurophysiol. 20, 408–434. 13439410 10.1152/jn.1957.20.4.408

[B33] Mountcastle V. B. (1997). The columnar organization of the neocortex. Brain 120, 701–722. 9153131 10.1093/brain/120.4.701

[B34] Nordlie E. Gewaltig M.-O. Plesser H. E. (2009). Towards reproducible descriptions of neuronal network models. PLoS Comput. Bio. 5:e1000456. 10.1371/journal.pcbi.1000456 19662159 PMC2713426

[B35] Oja E. (1982). A simplified neuron model as a principal components analyzer. J. Math. Biol. 15, 267–273. 7153672 10.1007/BF00275687

[B36] Paullus J. R. Hickmott P. W. (2011). Diverse excitatory and inhibitory synaptic plasticity outcomes in complex horizontal circuits near a functional border of adult neocortex. Brain Res. 1416, 10–25. 10.1016/j.brainres.2011.07.062 21890112

[B37] Petersen R. S. Diamond M. E. (2000). Spatial-temporal distribution of whisker-evoked activity in the rat somatosensory cortex and the coding of stimulus location. J. Neurosci. 20, 6135–6143. 10934263 10.1523/JNEUROSCI.20-16-06135.2000 PMC6772590

[B38] Qi H. X. Kass J. H. Reed J. L. (2014). The reactivation of somatosensory cortex and behavioral recovery after sensory loss in mature primates. Front. Syst. Neurosci. 8:84. 10.3389/fnsys.2014.00084 24860443 PMC4026759

[B39] Recanzone G. H. Merzenich M. M. Jenkins W. M. Grajski K. A. Dinse H. R. (1992). Topographic reorganization of the hand representation in cortical area 3b of owl monkeys trained in a frequency-discrimination task. J. Neurophys. 67, 1031–1056. 1597696 10.1152/jn.1992.67.5.1031

[B40] Simons D. J. Carvell G. E. (1989). Thalamocortical response transformation in the rat vibrissa/barrel system. J. Neurophysiol. 61, 311–330. 2918357 10.1152/jn.1989.61.2.311

[B41] Stavrinou M. L. Della Penna S. Pizzella V. Torquati K. Cianflone F. Franciotti R.. (2007). Temporal dynamics of plastic changes in human primary somatosensory cortex after finger webbing. Cereb. Cortex 17, 2134–2142. 10.1093/cercor/bhl120 17110591

[B42] Sun Q. Huguenard J. R. Prince D. A. (2006). Barrel cortex microcircuits: thalamocortical feedforward inhibition in spiny stellate cell is mediated by a small number of fast-spiking interneurons. J. Neurosci. 26, 1219–1230. 10.1523/JNEUROSCI.4727-04.2006 16436609 PMC6674555

[B43] Sur M. (1980). Receptive fields of neurons in areas 3b and 1 of somatosensory cortex in monkeys. Brain Res. 198, 465–471. 6250672 10.1016/0006-8993(80)90762-3

[B44] Sur M. Garraghty P. E. Bruce C. J. (1985). Somatosensory cortex in macaque monkeys: laminar differences in receptive field size in areas 3b and 1. Brain Res. 342, 391–395. 4041845 10.1016/0006-8993(85)91144-8

[B45] Sur M. Merzehich M. M. Kaas J. H. (1980). Magnification, receptive field area, and “hypercolumn” size in areas 3b and 1 of somatosensory cortex in owl monkeys. J. Neurophysiol. 44, 295–311. 7411189 10.1152/jn.1980.44.2.295

[B46] Takeuchi A. Amari S. (1979). Formation of topographic maps and columnar microstructures in nerve fields. Biol. Cybern. 35, 63–72. 518933 10.1007/BF00337432

[B47] van Westen D. Fransson P. Olsrud. J Rosén. B Lundborg. G Larsson. E. M (2004). Fingersomatotopy in area 3b: an fMRI study. BMC Neurosci. 5:28. 10.1186/1471-2202-5-28 15320953 PMC517711

[B48] Vogels T. P. Froemke R. C. Doyon N. Gilson M, Haas, J. S. Liu R.. (2013). Inhibitory synaptic plasticity: spike timing-dependence and putative network function. Front. Neural Circuits 7:119. 10.3389/fncir.2013.00119 23882186 PMC3714539

[B49] von der Malsburg C. (1973). Self-organization of orientation sensitive cells in the striate cortex. Kybernetik 14, 85–100. 4786750 10.1007/BF00288907

[B50] Willshaw D. J. von der Malsburg C. (1976). How patterned neural connections can be set up by self-organization. Proc. R. Soc. Lond. B 194, 431–445. 12510 10.1098/rspb.1976.0087

[B51] Wilson H. R. Cowan J. D. (1972). Interactions in localized populations of model neurons. Biophys. J. 12, 1–24. 4332108 10.1016/S0006-3495(72)86068-5 PMC1484078

[B52] Wilson H. R. Cowan J. D. (1973). A mathematical theory of the functional dynamics of cortical and thalamic nervous tissue. Kybernetik 13, 55–80. 4767470 10.1007/BF00288786

[B53] Zimmerman A. Bai L. Ginty D. D. (2014). The gentle touch receptors of mammalian skin. Science 346, 950–954. 25414303 10.1126/science.1254229 PMC4450345

